# Tunable plasmonic core–shell heterostructure design for broadband light driven catalysis†
†Electronic supplementary information (ESI) available: Experimental details, 15 figures and 4 tables. See DOI: 10.1039/c8sc04479a


**DOI:** 10.1039/c8sc04479a

**Published:** 2018-11-15

**Authors:** Chuang Han, Shao-Hai Li, Zi-Rong Tang, Yi-Jun Xu

**Affiliations:** a State Key Laboratory of Photocatalysis on Energy and Environment , College of Chemistry , Fuzhou University , Fuzhou , 350116 , China . Email: yjxu@fzu.edu.cn; b College of Chemistry , New Campus , Fuzhou University , Fuzhou , 350116 , China

## Abstract

Considerable effort has been devoted to manipulating the optical absorption of metal nanostructures for diverse applications. However, it still remains a challenge to develop a general and flexible method to promote broadband absorption of metal nanostructures without changing their size and shape. Here, we report a new strategy of hybridizing two conceptually different optical models to realize broadband absorption enhancement of metal nanoparticles (NPs), which is enabled by constructing a core–shell heterostructure, consisting of a spherical dielectric core covered by a metal NPs interlayer and tunable semiconductor shell. This approach integrates the interfacial photon management, photoexcitation of metal NPs and injection of hot charge carriers into the semiconductor shell, and results in distinctly enhanced hot charge carrier generation and transfer, thereby boosting the broad-spectrum light driven catalysis. The structure–plasmon–catalysis interplay of the heterostructure is comprehensively studied and optimized. This proof-of-concept proves to be generally feasible by varying the type of both metal NPs and support medium, opening a new avenue to control the optoelectronic properties of materials.

## Abstract 2

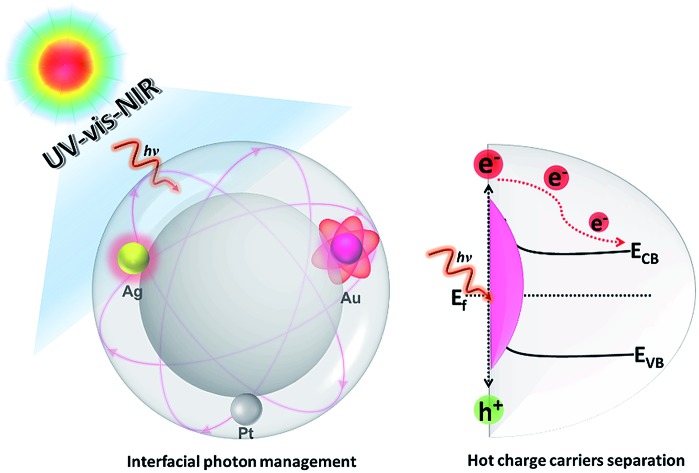
A tunable core–shell heterostructure design coupling two conceptually different optical absorption models for improved broadband light absorption and hot charge carrier separation toward plasmon-mediated photocatalysis.

## Introduction

Surface plasmon resonance (SPR), resulting from the collective oscillations of delocalized electrons in a metal particle,^1^–^4^ offers great opportunity to design tailored light absorption materials for solar-to-chemical (or electric) energy conversion without the bandgap limitations of traditional semiconductor materials.^5^–^10^ However, the inherent drawbacks such as the limited spectral range of low-loss plasmon resonance, weak light absorption intensity and rapid hot charge carrier relaxation fundamentally restrict the solar energy conversion efficiency of plasmonic metals.^7^–^9^,^11^ Various strategies have been developed to tune the optical absorption and prolong the lifetime of hot charge carriers of plasmonic metals, thereby boosting the efficiency of photoredox reactions.^7^–^9^,^12^–^17^ Notably, these strategies have predominantly relied on altering the nature (*e.g.*, size, shape or type) of metal nanostructures and coupling with other metals to maximize light–matter interaction and energy transfer. It is still challenging to develop a general and cost-efficient method to simultaneously promote the broad-spectrum light absorption and hot charge carrier separation and transfer of metal nanostructures, but without synthetically changing their size and shape.

Light scattering by dielectric spherical particles gives rise to electronic field enhancement and photon confinement at the interface that can be used to enhance the light harvesting efficiency.^18^–^20^ Theoretical studies have predicted that when near-field scattering radiation overlaps with SPR, hybrid resonance modes generally occur and specific optical absorption enhancement will emerge.^21^,^22^ Yet, it still remains unknown whether the combination of these two conceptually different strategies, *i.e.*, expanding the light response region by the SPR effect and enhancing light absorption intensity with a near-field scattering optical model, could be expected to combinatively manipulate interfacial photon interaction, enhance the light harvesting capability of plasmonic metal NPs in a broadband spectral range, and result in the boosted generation and separation of hot charge carriers for improved photoredox catalysis performance.

Since both near-field scattering and SPR are greatly dependent on the geometrical arrangement of the building units,^5^,^23^ a systematic study on these two optical cooperative effects will lead us to ultimately unveil the whole scenario of how the heterointerface and compositions of metal–dielectric sphere hybrid structures affect the light absorption properties of plasmonic metal NPs and the corresponding photocatalytic performance triggered by light-excited hot charge carriers from plasmonic metal NPs, which will further guide the design and optimization of the plasmonic photocatalyst.

Herein, we report a general and cost-efficient paradigm of core–shell heterostructure design for broadband light absorption management of plasmonic metal NPs without changing their size and shape. This approach mainly involves construction of a three dimensional (3D) core–shell nanostructure consisting of a spherical SiO_2_ core covered by a plasmonic Au NPs interlayer and tunable TiO_2_ semiconductor shell. In such a heterostructure, the multiple light–matter interactions at the core–shell interfaces enable tuning and enhancing the optical absorption of plasmonic Au NPs across the ultraviolet-visible-near infrared (UV-vis-NIR) region. The plasmonic Au NPs can harvest broadband light to generate and inject hot charge carriers into TiO_2_ for driving surface redox reactions. The light absorption and photoactivity enhancement is highly dependent on the diameter of the spherical dielectric core, the type of metal NPs and the thickness of the semiconductor shell. The generality of this strategy has been proven by varying both the metal NPs (plasmonic Ag or nonplasmonic Pt NPs) and support medium (*e.g.*, TiO_2_, ZnO, and organic polymer polystyrene) of the core–shell composite. Our work suggests promising scope to adopt this new methodology of coupling the SPR model of a plasmonic metal with a near-field scattering optical model to control the structure–plasmon–catalysis interplay of plasmonic metal nanostructures, thereby realizing efficient broadband light driven catalysis.

## Experimental section

### Preparation

Au–SiO_2_ composites with different weight contents of Au were prepared by a facile electrostatic self-assembly method using negatively charged citrate-stabilized Au NPs and positively charged 3-aminopropyl-triethoxysilane (APTES)-functionalized SiO_2_ spheres in the aqueous phase.^18^,^24^ The Au–SiO_2_@TiO_2_ composites were synthesized by coating a TiO_2_ shell on the surface of Au–SiO_2_ using a cooperative assembly-directed procedure.^25^ For more experimental details see the Methods in the ESI.†

### Characterization

Zeta potential (*ξ*) measurements of the samples were performed by dynamic light scattering analysis (Zeta sizer 3000HSA) at room temperature. The optical properties of the samples were measured by ultraviolet-visible-near infrared (UV-vis-NIR) diffuse reflectance spectroscopy (DRS) on a UV-vis Spectrophotometer (Thermo Scientific Evolution 200 Series) with BaSO_4_ as the internal reflectance standard. The content of Au was measured by inductively coupled plasma mass spectrometry (ICP-MS, XSERISE 2). The morphology and elemental distribution of the samples were analyzed by field-emission scanning electron microscopy (FESEM) on a FEI Nova NANOSEM 230 spectrophotometer and transmission electron microscopy (TEM), high-resolution TEM (HRTEM), energy dispersive X-ray spectroscopy (EDX) and elemental mapping analysis using a JEOL model JEM 2010 EX instrument at an accelerating voltage of 200 kV. Micromeritics ASAP2010 equipment was used to determine the nitrogen adsorption–desorption isotherms and the Brunauer–Emmett–Teller (BET) surface areas. The sample was degassed at 140 °C for 5 h and then analyzed at –196 °C. The transient absorption (TA) data were measured by using femtosecond transient absorption spectroscopy (Time-Tech Spectra, femtoTA-100). Part of the 800 nm output pulse from the amplifier was used to pump a TOPAS Optical Parametric Amplifier (OPA) which generates a 400 nm pump beam. The pump pulses were chopped with a synchronized chopper at 500 Hz and the absorbance change was calculated with two adjacent probe pulses (pump-blocked and pump-unblocked), and the pump pulse power was approximately 70 μJ cm^–2^. The samples were dispersed in ethylene glycol for all pump-probe characterization experiments performed under ambient conditions. The electrochemical analysis was carried out in a conventional three-electrode cell using a Pt plate and an Ag/AgCl electrode as the counter electrode and reference electrode, respectively. The working electrode was prepared on fluorine-doped tin oxide (FTO) glass that was cleaned by sonication in ethanol for 30 min and dried at 80 °C. The boundary of the FTO glass was protected using Scotch tape. A 10 mg sample was dispersed in 1 mL of DMF by sonication to get a slurry. The slurry was spread onto the pretreated FTO glass. After air drying, the working electrode was further dried at 120 °C for 2 h to improve adhesion. Then, the Scotch tape was unstuck, and the uncoated part of the electrode was isolated with epoxy resin. The exposed area of the working electrode was 0.25 cm^2^. The photocurrent density was measured on a BAS Epsilon workstation without bias. The electrolyte was 0.2 M Na_2_SO_4_ aqueous solution. Incident photon-to-current conversion efficiency (IPCE) was measured by using a PEC-S20 (Peccell Technology Co. Ltd.) without bias potential. The IPCE value was calculated according to the following equation:^11^,^18^ 
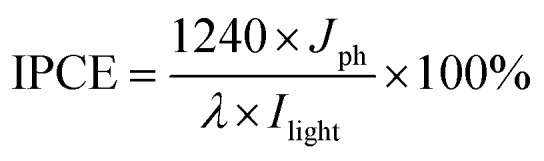
where *J*_ph_ is the photocurrent density, *λ* is the incident light wavelength, and *I*_light_ is the incident light power density for each wavelength.

### Photoactivity testing

In a typical procedure for photocatalytic reduction of nitro compounds, a 300 W Xe arc lamp (PLS-SXE 300, Beijing Perfect light Co., Ltd.) with a filter (Shanghai Mega-9 Optoelectronic Co., Ltd.) to cut off the light of wavelengths *λ* < 410 nm was used as the irradiation source. The light intensity was fixed at 800 mW cm^–2^. 20 mg Au–SiO_2_ or 25 mg core–shell Au–SiO_2_@TiO_2_ and 80 mg of ammonium formate (as a quencher for photogenerated holes) were added to 40 mL of the nitroaromatic solution (5 mg L^–1^) in a quartz vial. Before light illumination, the above suspension was stirred in the dark for 1 h to ensure the establishment of adsorption–desorption equilibrium between the sample and reactant. During the process of the reaction, 2 mL of sample solution was collected at a certain time interval and centrifuged to remove the catalyst completely at 12 000 rpm. Afterward, the solution was analyzed on a Varian UV-vis Spectrophotometer. The whole experimental process was conducted at room temperature under N_2_ bubbling at a flow rate of 80 mL min^–1^. The photodeposition of Ag was conducted under identical conditions except that nitroaromatic solution was replaced by 40 mL of 0.01 M AgNO_3_ solution. In photocatalytic oxidation of alcohols, a mixture of an alcohol (0.1 mmol) and photocatalyst was dispersed in oxygen-saturated benzotrifluoride (BTF; 1.5 mL). The mixture was transferred into a 10 ml Pyrex glass bottle filled with molecular oxygen at a pressure of 0.1 MPa and stirred for half an hour to blend the catalyst evenly into the solution. The suspensions were irradiated with visible-near infrared (vis-NIR, 410 nm < *λ* < 1100 nm) light. After the reaction, the product and remaining substrate were analyzed with an Agilent Gas Chromatograph (GC-7820). To obtain the action spectrum, the reaction system was continuously illuminated under nearly monochromatic light with a light intensity of 40 mW cm^–2^ for 10 h. The recycling testing and control experiments were conducted under identical conditions to those of the photocatalytic reaction as mentioned above. The conversion (%) of the aromatic nitro compounds and benzylic alcohols during the photocatalytic reaction is reported as (*C*_0_ – *C*)/*C*_0_ × 100%, where *C*_0_ is the initial concentration of the reactant after the establishment of adsorption desorption equilibrium, and *C* is the concentration of the reactant at a certain time interval after the photocatalytic reaction.

## Results and discussion

### Synthesis and morphology analysis

**Fig. 1:**
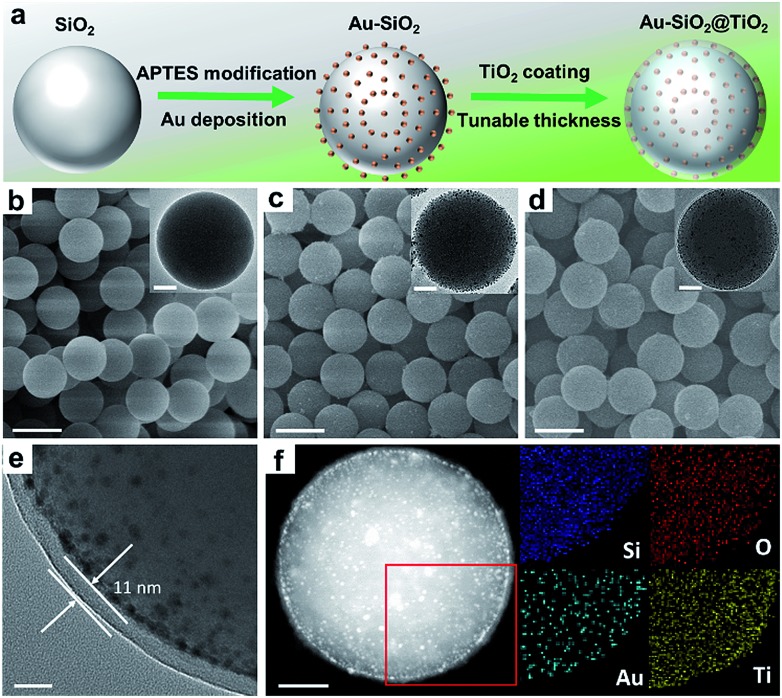
(a) Schematic illustration of the fabrication process of the Au–SiO_2_@TiO_2_ composite. Scanning electron microscopy (SEM) images of (b) bare SiO_2_, (c) 5% Au–SiO_2_ and (d) 5% Au–SiO_2_@TiO_2_. Scale bar, 500 nm. The insets in (b–d) show the transmission electron microscopy (TEM) images of the corresponding samples. Scale bar, 100 nm. (e) TEM image of 5% Au–SiO_2_@TiO_2_ with a shell thickness of 11 nm. Scale bar, 20 nm. (f) High-angle annular dark-field scanning transmission electron microscopy (HAADF-STEM) image (left) and elemental mapping results for the boxed area in the main image of 5% Au–SiO_2_@TiO_2_ with a shell thickness of 11 nm. Scale bar, 100 nm.

Efficient harvesting of solar energy to generate hot charge carriers requires that the plasmonic metal nanostructures have strong and broadband light absorption.^26^ Here, we achieve the broadband optical absorption of plasmonic metal NPs by adjusting their dielectric environment, while preserving the size of metal NPs. Taking the plasmonic Au NPs as an example, the generic procedure mainly involves uniform self-assembly of Au NPs onto the surface of 3-aminopropyl-triethoxysilane (APTES)-modified SiO_2_ spheres to synthesize Au–SiO_2_ by electrostatic attraction (Fig. S1a†), and subsequently coating a tunable thin-layer of TiO_2_ over Au–SiO_2_ using a cooperative assembly-directed process,^25^ as illustrated in Fig. 1a. The morphologies of SiO_2_ and Au–SiO_2_ are shown in Fig. 1b and c, respectively. As compared to the smooth surface of bare SiO_2_ spheres (Fig. 1b), the anchored Au NPs with a mean size of 5.5 nm (Fig. S1b and S1c†) can be clearly observed on the surface of Au–SiO_2_ (Fig. 1c). The intimate interfacial contact between SiO_2_ and Au NPs (Fig. S1d†) assured by surface modification of SiO_2_ through APTES allows an efficient light–matter interaction between them.^18^

For the Au–SiO_2_@TiO_2_ composite, the scanning electron microscopy (SEM) and transmission electron microscopy (TEM) images (Fig. 1d and e) indicate that Au–SiO_2_ is well covered by the TiO_2_ shell with an average thickness of 11 nm. Notably, the developed cooperative assembly-directed method enables us to tune the shell thickness by controlling the additive volume of the titanium precursor (for more details, see the Methods in the ESI and Fig. S2†).^25^ The Au NPs embedded into the TiO_2_ shell with a similar size and shape to those in Au–SiO_2_ are clearly distinguished. The Au–SiO_2_@TiO_2_ composite also exhibits a spherical shape, indicating a uniform coating layer of TiO_2_ over the entire surface of Au–SiO_2_. Energy dispersive X-ray (EDX) elemental mapping in Fig. 1f shows the spatial distributions of Si, O, Au and Ti elements, in which the spatial distribution ranges of Ti and O are larger than that of Au and Si, indicating that the TiO_2_ thin-layer is coated on the outermost surface of Au–SiO_2_ to form such a 3D core–shell structured Au–SiO_2_@TiO_2_ composite.

### Interface-induced broadband optical absorption

**Fig. 2:**
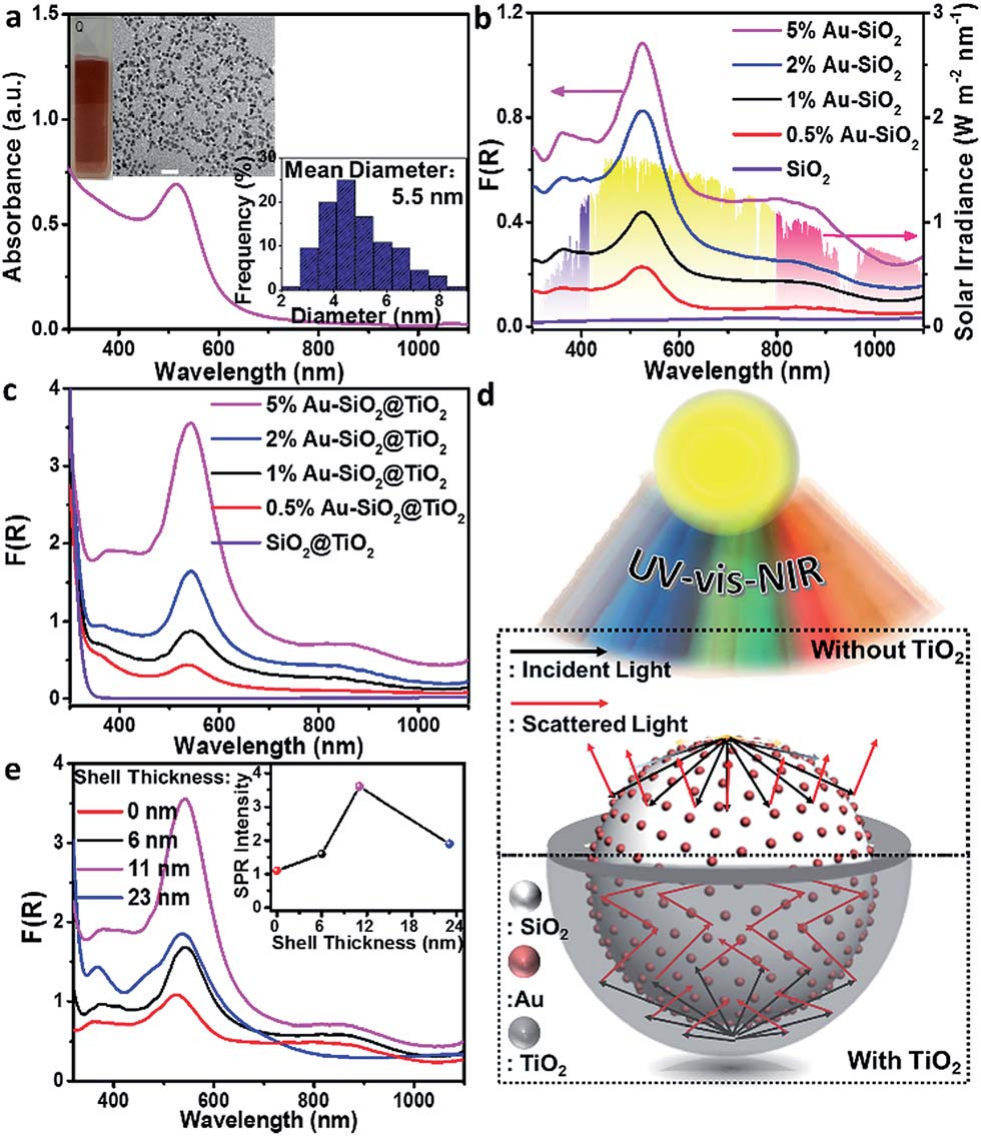
(a) Ultraviolet-visible-near infrared (UV-vis-NIR) absorption spectrum of Au NPs. The insets in (a) show the photograph, transmission electron microscopy (TEM) image and size distribution histogram of Au NPs colloids. Scale bar, 20 nm. UV-vis-NIR diffuse reflectance spectra (DRS) of (b) Au–SiO_2_ and (c) Au–SiO_2_@TiO_2_ with a shell thickness of 11 nm with different weight contents of Au. (d) Schematic illustration of the interfacial photon management in Au–SiO_2_ and Au–SiO_2_@TiO_2_. (e) UV-vis-NIR DRS of 5% Au–SiO_2_@TiO_2_ with different shell thicknesses. The inset in (e) shows the surface plasmon resonance (SPR) absorption intensity as a function of the average shell thickness of Au–SiO_2_@TiO_2_. The diameter of SiO_2_ is 450 nm.

To verify the enhancement of light harvesting in this core–shell structure model, the optical properties of the samples were characterized. As shown in Fig. 2a, the bare aqueous Au NPs with a mean size of 5.5 nm exhibit a single SPR absorption centered at 515 nm. When these Au NPs are assembled onto the surface of SiO_2_ spheres (with a diameter of about 450 nm), three distinct absorption modes located at ∼360, 525 and 840 nm appear in the UV-vis-NIR diffuse reflectance spectra (DRS) of Au–SiO_2_, resulting in a large spectral overlap with the solar irradiance in the visible-near infrared (vis-NIR) region (Fig. 2b). The peak at 525 nm is attributed to the characteristic SPR absorption of Au NPs. Compared to the aqueous dispersion of Au NPs, the SPR absorption peak in Au–SiO_2_ is red-shifted by 10 nm due to the higher refractive index of SiO_2_ (*n* = 1.55) than that of water (*n* = 1.33).^27^,^28^ The other two absorption peaks cannot be detected for either bare SiO_2_ spheres or Au NPs aqueous dispersion. In addition, with the increase of the Au weight content in the Au–SiO_2_ composite, the intensities of these absorption peaks gradually enhance while their peak positions almost remain unchanged, thereby indicating that these characteristic peaks of Au–SiO_2_ are ascribed to the near-field scattering light–matter interaction between SiO_2_ spheres and Au NPs,^18^,^29^ rather than the interparticle coupling effect or aggregation of Au NPs as reported by previous studies.^28^,^30^–^32^

DRS spectra of the Au–SiO_2_@TiO_2_ composites with different Au weight contents are shown in Fig. 2c, where three well-defined absorption peaks at ∼365, 540 and 880 nm can be distinguished. As compared to Au–SiO_2_ containing the same amount of Au NPs, these peaks are slightly red-shifted and greatly intensified due to the coating of the TiO_2_ shell.^18^,^21^,^33^ The intensity of SPR absorption located at ∼540 nm increases almost linearly with increasing the weight content of Au in the Au–SiO_2_@TiO_2_ composite, as shown in Fig. S3.† These results indicate that the TiO_2_ shell with a high refractive index (*n* = 2.49) could serve as an effective reflecting layer to modify internal light reflection and greatly increase the chance of Au NPs to absorb photons, as demonstrated in Fig. 2d.^20^,^33^–^35^ We also noted that, apart from the peak at ∼540 nm ascribed to the SPR absorption of Au NPs, the other two peaks have not been observed in previously reported Au NPs–semiconductor composites.^24^,^33^,^36^–^40^ In addition, no distinct absorption peak can be observed for SiO_2_@TiO_2_, except the intrinsic exciton absorption edge of TiO_2_. These results reveal that the proper control of the configurations such as the internal structure organization and shell thickness is crucial to achieve the broad-spectrum light absorption of the Au–SiO_2_@TiO_2_ composite. The tunable core–shell ensemble provides a flexible platform for investigating the influence of these factors on the photoabsorption of plasmonic Au NPs.

We then explored the effects of TiO_2_ shell thickness on the photoabsorption of supported Au NPs. DRS spectra of Au–SiO_2_@TiO_2_ with different thicknesses of the TiO_2_ shell (Fig. 2e) suggest that the peak positions of resonance absorption are not sensitive to the change in TiO_2_ shell thickness, while the peak intensity exhibits hump-like dependence on the coating thickness of the TiO_2_ shell, as shown in the inset of Fig. 2e. With increasing the thickness of the TiO_2_ shell, the absorption intensity initially enhances and reaches a maximum when the thickness of TiO_2_ is 11 nm. Excessive coating of the TiO_2_ shell will enhance the external photon reflection by TiO_2_ rather than photon harvesting of Au NPs at the core–shell interface, due to the relatively large size of the core–shell composite,^40^–^42^ which finally leads to the decrease of absorption intensity and even vanishing of the near-field scattering-mediated absorption model.

Next, we investigated the effects of the internal core on tuning the optical absorption of Au NPs by varying the diameter of SiO_2_ spheres (Fig. S4†) from 150 nm to 600 nm. It can be seen from the DRS results in Fig. S5† that small-sized SiO_2_ spheres (with a diameter < 350 nm) have only affected the photoabsorption of Au NPs in the ultraviolet region. As the size of the SiO_2_ sphere becomes larger, more absorption modes are observed within the UV-vis-NIR region. When the diameter of the SiO_2_ sphere is larger than 400 nm, the Au–SiO_2_ sample exhibits a new absorption band in the near infrared (NIR) region. In addition, the absorption maxima of Au–SiO_2_ show an obvious red-shift along with the increment of the SiO_2_ diameter. Generally, for Au NPs, only those larger than 50 nm or large-sized aggregates can disclose a noticeable NIR absorption band.^30^,^43^,^44^ Our results indicate that by merely modulating the diameter of SiO_2_ supports, the absorption spectrum of small Au NPs (<10 nm) supported on spherical SiO_2_ can be delicately tuned to the NIR region. This result inspires us to find out whether it is possible to tune the optical properties of Au NPs by using other types of spherical supports. In this context, we further studied the interfacial photon interaction between Au NPs and a series of other dielectric spheres including inorganic semiconductors (*e.g.*, TiO_2_ and ZnO) and organic polymer polystyrene (PS). UV-vis-NIR DRS spectra (Fig. S6†) of these composites suggest that the self-assembly of an Au NPs antenna layer onto a spherical dielectric support is a general method to maneuver the photoabsorption of Au NPs without changing their size.

To uncover the synergistic interaction between metal NPs and the spherical core and understand the fundamental role of metal NPs played in this model system, other metal NPs such as Ag and Pt NPs (Fig. S7†) were also loaded onto the spherical supports (SiO_2_, TiO_2_, ZnO and PS) by using the same self-assembly approach. The UV-vis-NIR DRS results in Fig. S8† reveal an analogous absorption enhancement with additional distinct absorption peaks on Ag or Pt NPs decorated SiO_2_ spheres and other dielectric spheres. In addition, the dielectric spheres display a strong resonance cooperative effect with plasmonic Au and Ag NPs,^21^,^23^ leading to their more efficient broadband light absorption than supported Pt NPs, especially in the visible-near infrared (vis-NIR) region (Fig. S8†). This confirms that the photoabsorption of both plasmonic and nonplasmonic metals can be tuned by interfacial photon management, which closely depends on the properties of the surrounding medium, such as the diameter of spherical supports, and the combination of the SPR effect with the near-field scattering optical model gives rise to more efficient light harvesting capability of metal NPs. By jointly studying the absorption modes of SiO_2_ supported Au, Ag, and Pt NPs (Fig. S9†), the variation trends of the absorption maxima with increasing the diameter of SiO_2_ spheres are summarized in Fig. S9c.† As is clearly seen, in order to generate an efficient cooperative effect between near-field scattering and SPR absorption of Au NPs, the diameter of SiO_2_ spheres should be set at ∼300 nm. With regard to producing distinct NIR light (*λ* > 800 nm) absorption peaks, the diameter of SiO_2_ spheres should be larger than 420 nm.

### Broadband light driven catalysis

**Fig. 3:**
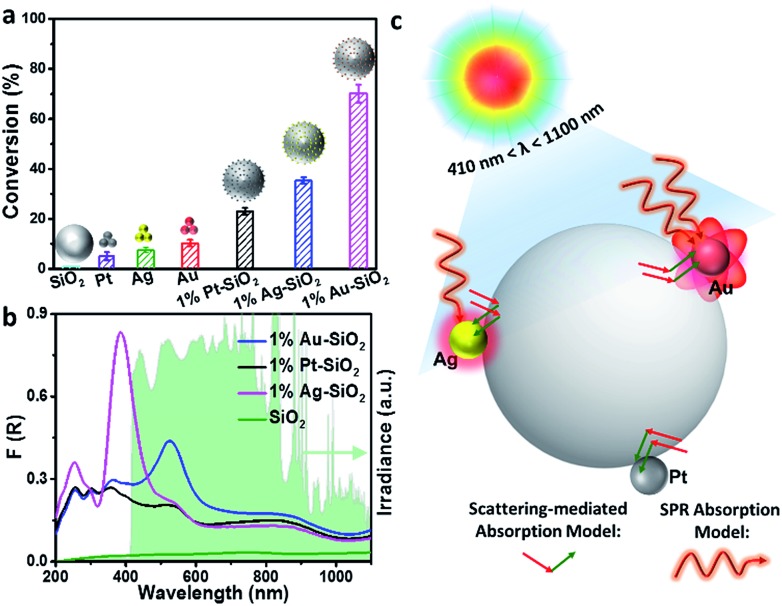
(a) Photoreduction of 4-nitroaniline (4-NA) over bare SiO_2_, metal NPs, and 1% M–SiO_2_ (M = Au, Ag, Pt) under visible-near infrared (vis-NIR) light (410 nm < λ < 1100 nm) irradiation for 5 h. The insets of (a) show the model of the corresponding samples. Error bars represent the standard deviation calculated from triplicate experiments. (b) Ultraviolet-visible-near infrared (UV-vis-NIR) diffuse reflectance spectra (DRS) of SiO_2_ and 1% M–SiO_2_. The green area in (b) shows the spectrum of the irradiation source. (c) Schematic showing the mechanism of broadband optical absorption enhancement in M–SiO_2_ composites. The diameter of SiO_2_ is 450 nm.

To detect whether the broad-spectrum light absorption is able to promote solar-to-chemical energy conversion, the photocatalytic performances of the samples were examined under longer-wavelength vis-NIR light (410 nm < *λ* < 1100 nm) irradiation. Fig. 3a shows the photoactivity of the bare SiO_2_, metal NPs, and SiO_2_ supported metal NPs (M–SiO_2_, M = Au, Ag or Pt) composites with the same SiO_2_ diameter of 450 nm toward photoreduction of 4-nitroaniline (4-NA) at room temperature and under anaerobic conditions. It is seen that the bare SiO_2_ shows negligible photoactivity because it has no light harvesting capability in the vis-NIR region (Fig. 3b). By contrast, 1% M–SiO_2_ exhibits obvious activity under the same conditions, among which 1% Au–SiO_2_ and 1% Ag–SiO_2_ samples disclose enhanced photoactivity as compared to bare Au, Ag, Pt and even 1% Pt–SiO_2_, due to their largely enhanced broad-spectrum light absorption in the vis-NIR spectral range (Fig. 3b) resulting from the combination of the near-field scattering-mediated optical absorption model with the SPR model (Fig. 3c). The main SPR absorption peak of 1% Ag–SiO_2_ is located in the ultraviolet region, which accounts for its lower activity than 1% Au–SiO_2_ under vis-NIR light irradiation. These results indicate that the concurrent optical models are able to cooperatively contribute to the activity enhancement of the plasmonic Au–SiO_2_ composite.

**Fig. 4:**
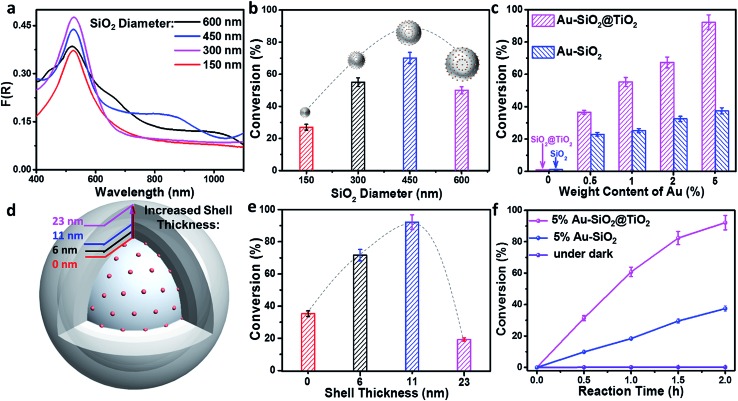
(a) Ultraviolet-visible-near infrared (UV-vis-NIR) diffuse reflectance spectra (DRS) of 1% Au–SiO_2_ with different diameters of SiO_2_ spheres. (b) Photoreduction of 4-nitroaniline (4-NA) over 1% Au–SiO_2_ with different diameters of SiO_2_ spheres under visible-near infrared (vis-NIR) light (410 nm < λ < 1100 nm) irradiation for 5 h. The insets of (b) show the model of the corresponding samples. (c) Photoreduction of 4-NA over Au–SiO_2_ and Au–SiO_2_@TiO_2_ with different weight contents of Au under vis-NIR light irradiation for 2 h. (d) Schematic model of 5% Au–SiO_2_@TiO_2_ with different shell thicknesses. (e) Photoreduction of 4-NA over 5% Au–SiO_2_@TiO_2_ with different shell thicknesses under vis-NIR light irradiation for 2 h. (f) Time-online profiles of photoreduction of 4-NA over optimal 5% Au–SiO_2_ and 5% Au–SiO_2_@TiO_2_ under vis-NIR light irradiation or over 5% Au–SiO_2_@TiO_2_ under dark conditions. Error bars represent the standard deviation calculated from triplicate experiments. The TiO_2_ shell thickness of 5% Au–SiO_2_@TiO_2_ is 11 nm.

The influence of SiO_2_ size on the photoactivity was studied for Au–SiO_2_ with four typical SiO_2_ diameters of 150, 300, 450 and 600 nm. According to Fig. S9c,† Au–SiO_2_ with a SiO_2_ diameter of 150 nm has a very weak interface-induced absorption enhancement in the vis-NIR region, whereas that with a SiO_2_ diameter of 300 nm possesses an efficient cooperative effect between near-field scattering and SPR absorption of Au NPs in the visible region. Further increasing the diameter of SiO_2_ to 450 or 600 nm leads to pronounced vis-NIR light absorption, but a weak cooperative effect. The above results can also be substantiated by the DRS spectra of the composites. As shown in Fig. 4a, the Au–SiO_2_ composite with a core diameter of 300 nm exhibits the strongest SPR absorption intensity among all the samples, but weaker NIR light absorption than Au–SiO_2_ with SiO_2_ diameters of 450 and 600 nm. The photocatalytic activities of the samples, as disclosed in Fig. 4b, show that Au–SiO_2_ with a SiO_2_ diameter of 450 nm exhibits the highest activity. Considering that all the samples contain the same weight content of Au (*i.e.*, 1%), the highest photocatalytic activity of Au–SiO_2_ with a SiO_2_ diameter of 450 nm can be attributed to the fact that this specific diameter of SiO_2_ enables the efficient photoabsorption of Au–SiO_2_ both in the SPR model and near-field scattering-mediated absorption model. Unless specifically mentioned, the diameter of SiO_2_ is fixed at 450 nm in the subsequent experiments.

The photoactivity of Au–SiO_2_ and Au–SiO_2_@TiO_2_ with different Au weight contents is shown in Fig. 4c. The conversion of 4-NA is gradually improved with increasing Au weight content. It is worth noting that due to the limitation of the electrostatic assembly method, the maximum load-bearing capacity of metal NPs is about 5% Au weight content over the surface of SiO_2_. Au–SiO_2_@TiO_2_ exhibits higher activity than Au–SiO_2_ with the same amount of Au loading. For example, 92% conversion of 4-NA is obtained over 5% Au–SiO_2_@TiO_2_ with vis-NIR light irradiation for 2 h, which is much higher than that of 5% Au–SiO_2_ (*ca.* 38%).

Since the bare SiO_2_ and SiO_2_@TiO_2_ core–shell composite show no photoactivity for the conversion of 4-NA (Fig. 4c), the higher photoactivity of Au–SiO_2_@TiO_2_ than Au–SiO_2_ should be closely linked with the interfacial interaction between the TiO_2_ shell and Au NPs layer. On the basis of such consideration, we examined the photoactivity of 5% Au–SiO_2_@TiO_2_ with different thicknesses of the TiO_2_ shell (*e.g.*, 6, 11 and 23 nm, Fig. 4d). The results (Fig. 4e) reveal that 5% Au–SiO_2_@TiO_2_ with a shell thickness of 11 nm exhibits the highest photoactivity among these samples. An excess amount of TiO_2_ coating in 5% Au–SiO_2_@TiO_2_ could lead to the hot charge carriers generated at the vicinity of Au NPs hardly reaching the TiO_2_ shell surface, where the redox catalysis reaction takes place,^21^ thus suppressing the photoactivity. Fig. 4f shows the time-online profiles of photoreduction of 4-NA over the optimal 5% Au–SiO_2_ and 5% Au–SiO_2_@TiO_2_. With extending the irradiation time, the conversion of 4-NA increases correspondingly. A control experiment for testing the activity of the 5% Au–SiO_2_@TiO_2_ sample in the dark shows no conversion of 4-NA. These results indicate that the reduction of 4-NA can be attributed to a characteristic photocatalytic process.^45^–^47^ The photoactivity enhancement of 5% Au–SiO_2_@TiO_2_ was also confirmed by the conversion of other aromatic nitro compounds (Table S1†) and benzylic alcohols (Table S2†) and photocatalytic CO_2_ reduction (Fig. S10a†) and water splitting (Fig. S10b†) under vis-NIR light irradiation, suggesting promising scope to adopt this core–shell ensemble for realizing broad-spectrum light driven artificial photosynthesis. In addition, the recycling photoactivity test indicates that the presence of the TiO_2_ shell can distinctly enhance the reusability and stability of the Au–SiO_2_@TiO_2_ photocatalyst during the reaction process (Fig. S11†).

### Mechanism of the photoactivity enhancement

**Fig. 5:**
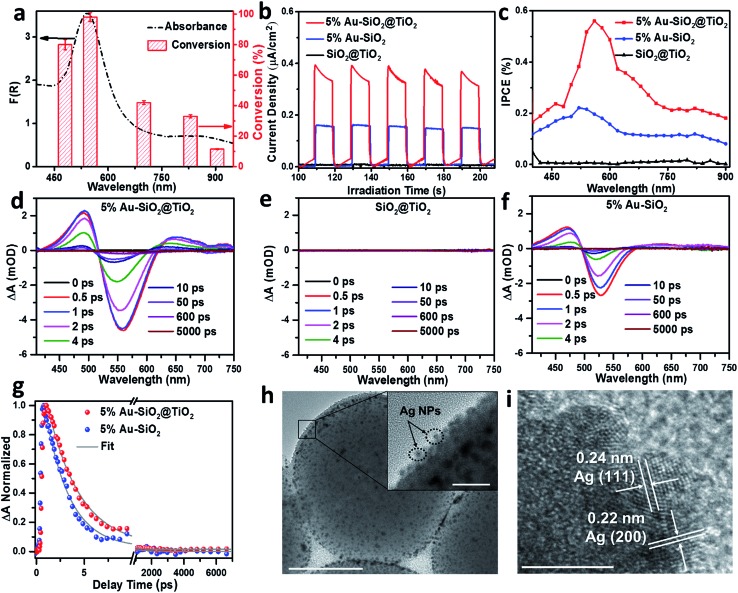
(a) Absorption spectrum (left axis) and action spectrum (right axis) of 5% Au–SiO_2_@TiO_2_ for the reduction of 4-nitroaniline (4-NA). The irradiation time with different monochromatic light is 10 h. The power density of different monochromatic light is 30.0 mW cm^–2^. Error bars represent the standard deviation calculated from triplicate experiments. (b) Transient photocurrent–time (*I*–*t*) curves under visible-near infrared (vis-NIR) light (410 nm < λ < 1100 nm) irradiation and (c) incident photon-to-current conversion efficiency (IPCE) of SiO_2_@TiO_2_, 5% Au–SiO_2_ and 5% Au–SiO_2_@TiO_2_ electrodes. Transient absorption (TA) spectra of (d) 5% Au–SiO_2_@TiO_2_, (e) SiO_2_@TiO_2_ and (f) 5% Au–SiO_2_ at indicated delay time windows after 400 nm excitation. (g) TA kinetics for 5% Au–SiO_2_@TiO_2_ and 5% Au–SiO_2_ after 400 nm excitation and detection at 524 nm. (h) Transmission electron microscopy (TEM) image of 5% Au–SiO_2_@TiO_2_ after photodeposition of Ag NPs. Scale bar, 200 nm. The boxed area in the inset of (h) indicates the presence of Ag NPs. Scale bar, 20 nm. (i) High-resolution TEM (HRTEM) image of 5% Au–SiO_2_@TiO_2_ after photodeposition of Ag NPs. Scale bar, 5 nm. The TiO_2_ shell thickness of 5% Au–SiO_2_@TiO_2_ is 11 nm.

Wavelength-dependent action spectrum analysis was performed to further verify the contribution of Au NPs to the photoactivity of Au–SiO_2_@TiO_2_. As shown in Fig. 5a, the action spectrum of the optimal 5% Au–SiO_2_@TiO_2_ sample correlates well with its broadband light absorption. The conversion of 4-NA reaches its maximum value with the irradiation of monochromatic light centered at 540 nm. When the light source is switched to 905 nm NIR light, the 5% Au–SiO_2_@TiO_2_ hybrid also exhibits considerable photoactivity (12% conversion of 4-NA). These results suggest that the observed activity of Au–SiO_2_@TiO_2_, even under NIR irradiation, is initiated by the specific optical absorption of Au NPs in the Au–SiO_2_@TiO_2_ composite.^14^,^18^,^46^,^48^ To identify the contribution of the interfacial photon scattering from the internal SiO_2_ to the promoted solar-to-chemical energy conversion over Au–SiO_2_@TiO_2_, we prepared an Au@TiO_2_ yolk–shell structure by removing the SiO_2_ core from Au–SiO_2_@TiO_2_ (Fig. S12†). The comparisons between their light absorption and photoactivity indicate that both the optical absorption and photoactivity of Au–SiO_2_@TiO_2_ are higher than those of Au@TiO_2_, which demonstrates that the interfacial photon scattering from the SiO_2_ core can intensify the light absorption of Au NPs to improve the efficiency of generation and separation of hot charge carriers to drive the photocatalytic reactions.

The photoelectrochemical (PEC) measurements under vis-NIR light (410 nm < *λ* < 1100 nm) irradiation also confirmed the enhanced solar energy conversion efficiency of Au–SiO_2_@TiO_2_. The photocurrent generated by optimal 5% Au–SiO_2_@TiO_2_ under vis-NIR light irradiation is almost 2 times as high as that of 5% Au–SiO_2_ (Fig. 5b), suggesting improved hot charge carrier generation and separation induced by the coated TiO_2_ shell.^37^ The SiO_2_@TiO_2_ electrode generates negligible photocurrent under identical conditions, since it cannot absorb vis-NIR light, which is in line with the photoactivity measurements (Fig. 4c). The incident photon-to-current efficiency (IPCE) value of 5% Au–SiO_2_@TiO_2_ is higher than that of 5% Au–SiO_2_ and SiO_2_@TiO_2_ in the entire vis-NIR range (Fig. 5c). The surface area and adsorption experimental analysis (Fig. S13 and Table S3†) suggest that the coating of the TiO_2_ shell has no significant effect on the adsorption capacity of Au–SiO_2_@TiO_2_ as compared to Au–SiO_2_. The above results, together with the optical properties of the samples, illustrate that the primary role of the TiO_2_ shell with an appropriate thickness is to further enhance the light harvesting capability of Au–SiO_2_ and it contributes to extracting hot electrons from photoexcited Au NPs, thereby leading to enhanced photoactivity.

Transient absorption (TA) spectroscopy was employed to measure the lifetime of the hot electrons, which in turn provides direct evidence for the electron transfer direction.^15^,^49^–^51^ Fig. 5d shows the absorption spectroscopy of optimal 5% Au–SiO_2_@TiO_2_ after 400 nm excitation, where a long-lived exciton state can be clearly observed at wavelengths between 410 and 520 nm and a bleach signal located at ∼560 nm also appears. Since no signal is observed for SiO_2_@TiO_2_ due to the wide bandgap of TiO_2_ and SiO_2_ (Fig. 5e), the observed transient absorption for 5% Au–SiO_2_@TiO_2_ can be assigned to the excited Au NPs.^49^ In comparison with 5% Au–SiO_2_ (Fig. 5f), the intensity of the absorption signal of 5% Au–SiO_2_@TiO_2_ is increased, indicating the enhanced optical absorption and generation efficiency of hot electrons, which can be assigned to the contribution from near-field interfacial photon scattering collaboratively tuned by the SiO_2_ core and TiO_2_ shell.^15^ To evaluate the decay kinetics of photogenerated electron–hole pairs in the Au NPs component, the TA traces were fitted using a biexponential function as displayed in Fig. 5g, and their lifetimes (*τ*_i_) and amplitudes (*A*_i_) are summarized in Table S4.† The TA signal relaxation is dominated by a fast decay due to the hot electron relaxations *via* electron–electron and electron–phonon scatterings, followed by a slower decay arising from the phonon–phonon and phonon–solvent interactions.^50^ The 5% Au–SiO_2_ sample exhibits fast decay with a time scale of 2.5 ps (94%), which is consistent with previous reports on colloidal Au.^49^,^50^ The electrons transferring from the excited Au to TiO_2_ delay the exciton recombination process in Au NPs, giving rise to a prolonged lifetime of hot electrons within 5% Au–SiO_2_@TiO_2_.^49^,^51^

Thus far, a mechanism for interfacial hot charge carrier generation and transfer involved in photocatalytic redox reactions has been proposed, as illustrated in Fig. S14.† When Au–SiO_2_@TiO_2_ with a core–shell structure is irradiated with vis-NIR light, the Au NPs can simultaneously absorb the incident light and the scattered light at the near-field of the SiO_2_ dielectric surface to generate charge carriers. The energetic hot electrons are transferred to the conduction band (*E*_CB_) of the TiO_2_ shell for driving the surface reduction reactions (*e.g.*, reduction of aromatic nitro compounds, H_2_ evolution from water splitting and reduction of CO_2_), while the holes located below the Fermi level (*E*_f_) of Au NPs are tunneled to the surface of TiO_2_ to react with the electron donors (*e.g.*, benzylic alcohols and hole scavengers).^6^,^18^,^52^,^53^ We also performed a probe experiment of adding silver nitrate into the aqueous Au–SiO_2_@TiO_2_ suspension to further verify the transfer of electrons from Au NPs to the TiO_2_ shell under vis-NIR light irradiation.^52^,^53^ We can clearly observe the Ag particles photodeposited at the external surface of the TiO_2_ shell from the TEM images (Fig. 5h and i). The lattice fringes with 0.22 and 0.24 nm spacing are indexed to the (200) and (111) crystal planes of Ag, respectively (Fig. 5i). In addition, the DRS spectrum of 5% Au–SiO_2_@TiO_2_ after photodeposition of Ag (Fig. S15†) shows a clear absorption increase in the range of 300 to 500 nm, which is due to the reduction of silver ions to metallic Ag on the surface of the TiO_2_ shell.^53^ This suggests that the hot electrons photoexcited from Au NPs are able to transfer to the TiO_2_ shell, thereby reducing silver nitrate to metallic Ag particles.

## Conclusions

In conclusion, we have demonstrated that the optical absorption and hot charge carrier transport of plasmonic metal NPs can be delicately engineered by constructing a 3D core–shell composite. The combination of a near-field scattering promoted optical absorption model with the SPR model allows tuning of the optical absorption of plasmonic metal NPs across the UV-vis-NIR region, and results in enhanced interfacial hot charge carrier generation and transfer from metal NPs. The dielectric environment significantly affects the light absorption, hot charge carrier injection and photoactivity of metal NPs. Considering the generality that optical properties can be tuned in a broad-spectral range in such a core–shell structure by varying both the support and metal NPs, this strategy by managing the interfacial photons offers a controllable and flexible platform to modulate the optoelectronic properties of materials for promising solar energy conversion.

## Conflicts of interest

There are no conflicts to declare.

## Supplementary Material

Supplementary information
