# Supplementary material for: Tunable plasmonic core–shell heterostructure design for broadband light driven catalysis
Source: Chem Sci. 2018 Nov 15;9(48):8914–22. doi: 10.1039/c8sc04479a (PMC6335626; doi:10.1039/c8sc04479a)
Supplement: Supplementary file 1 [file SC-009-C8SC04479A-s001.pdf]

Electronic Supplementary Material (ESI) for Chemical Science.
This journal is © The Royal Society of Chemistry 2018

Electronic Supplementary Information

Tunable plasmonic core-shell heterostructure design for broadband light driven catalysis

Chuang Han,[ab] Shao-Hai Li,[ab] Zi-Rong Tang[b] and Yi-Jun Xu*[ab]

[a] State Key Laboratory of Photocatalysis on Energy and Environment, College of Chemistry, Fuzhou University, Fuzhou, 350116, P. R. China

[b] College of Chemistry, New Campus, Fuzhou University, Fuzhou, 350116, P. R. China

*Corresponding Author. Tel./Fax: +86 591 22865836; E-mail: yjxu@fzu.edu.cn

Contents list

Methods

Fig. S1 (a) Zeta potential of the Au NPs and APTES-SiO_2. (b) TEM image of 5% Au-SiO_2. (c) Size distribution histogram of Au NPs. (d) HRTEM image of the 5% Au-SiO_2 composite.

Fig. S2 (a) SEM image and (b and c) TEM images of 5% Au-SiO_2@TiO_2 with TiO_2 shell thickness of 6 nm. (d) SEM image and (e and f) TEM images of 5% Au-SiO_2@TiO_2 with TiO_2 shell thickness of 11 nm. (g) SEM image and (h and i) TEM images of 5% Au-SiO_2@TiO_2 with TiO_2 shell thickness of 23 nm.

Fig. S3 Plot of SPR absorption intensity of Au-SiO_2 and 5% Au-SiO_2@TiO_2 versus weight loading of Au NPs.

Fig. S4 SEM images of SiO_2 spheres with different average diameters of (a) 300 nm, (b) 350 nm, (c) 400 nm, (g) 450 nm, (h) 500 nm, (i) 600 nm and (d-f and j-l) the corresponding diameter distribution histograms.

Fig. S5 UV-vis-NIR DRS of 1% Au-SiO_2 with different diameters of SiO_2 spheres.

Fig. S6 SEM images of (a) TiO_2 spheres, (b) ZnO spheres and (c) PS spheres. UV-vis-NIR DRS of (d) Au-TiO_2 samples, (e) Au-ZnO samples and (f) Au-PS samples with different weight content of Au.

Fig. S7 UV-vis-NIR absorption spectrum of (a) Ag NPs and (b) Pt NPs.

Fig. S8 UV-vis-NIR DRS of (a) SiO_2 and 1% M-SiO_2, (b) TiO_2 and 1% M-TiO_2, (c) ZnO and 1% M-ZnO, (d) PS and 1% M-PS.

Fig. S9 UV-vis-NIR DRS of (a) 1% Au-SiO_2, (b) 1% Pt-SiO_2 and 1% Ag-SiO_2. (c) Absorption spectra of Au NPs and absorption maxima of 1% M-SiO_2 with different diameter of SiO_2 spheres.

Table S1 Photocatalytic reduction of a serious of nitroaromatics over the 5% Au-SiO_2 and 5% Au-SiO_2@TiO_2 under vis-NIR light irradiation.

Table S2 Photocatalytic oxidation of a range of benzylic alcohols over the 5% Au-SiO_2 and 5%

$Au-SiO_2@TiO_2$ under vis-NIR light irradiation.
Fig. S10 Photocatalytic (a) reduction of CO_2 and (b) water splitting over the 5% $Au-SiO_2$ and 5% $Au-SiO_2@TiO_2$ under vis-NIR light irradiation.
Fig. S11 Recycling tests for photocatalytic reduction of 4-NA over (a) 5% $Au-SiO_2@TiO_2$ and (b) 5% $Au-SiO_2$ under vis-NIR light irradiation. TEM images of (c) 5% $Au-SiO_2@TiO_2$ and (d) 5% $Au-SiO_2$ after recycling photocatalytic reactions.
Fig. S12 (a) TEM image of $Au@TiO_2$. (b) UV-vis-NIR DRS of $Au-SiO_2@TiO_2$ and $Au@TiO_2$. Photocatalytic performance of $Au-SiO_2@TiO_2$ and $Au@TiO_2$ for (c) reduction of 4-NA and (d) oxidation of BA under vis-NIR light irradiation.
Fig. S13 (a) Column plots showing the remaining 4-NA and BA in solution after being kept in the dark for 1 h to achieve the adsorption-desorption equilibrium over 5% $Au-SiO_2$ and 5% $Au-SiO_2@TiO_2$. (b) Nitrogen adsorption-desorption isotherms of 5% $Au-SiO_2$ and 5% $Au-SiO_2@TiO_2$.
Table S3 Summary of surface area and pore volume of the 5% $Au-SiO_2$ and 5% $Au-SiO_2@TiO_2$.
Table S4 Lifetime (τ_i) and amplitudes (A_i) of TA decays dynamics for 5% $Au-SiO_2$ and 5% $Au-SiO_2@TiO_2$.
Fig. S14 Schematic illustration for photogenerated charge carriers transfer of $Au-SiO_2@TiO_2$ under vis-NIR light irradiation.
Fig. S15 UV-vis-NIR DRS of 5% $Au-SiO_2@TiO_2$ before and after photodeposition of Ag.
References

Methods

Materials: Chloroauric acid ($HAuCl_4 \cdot 3H_2O$), silver nitrate ($AgNO_3$), hexachloroplatinic acid hexahydrate ($H_2PtCl_6 \cdot 6H_2O$), trisodium citrate dehydrate ($C_6H_5Na_3O_7 \cdot 2H_2O$), sodium borohydride ($NaBH_4$), tetraethyl orthosilicate ($C_8H_{20}O_4Si$, TEOS), isopropanol (C_3H_8O), ammonium hydroxide ($NH_3 \cdot H_2O$), anhydrous ethanol (C_2H_6O), 3-aminopropyl-triethoxysilane ($C_9H_{23}NO_3Si$, APTES), acetonitrile (C_2H_3N), benzyl alcohol (C_7H_8O, BA), 4-nitroaniline ($C_6H_6N_2O_2$, 4-NA), N,N-dimethylformamide (C_3H_7NO, DMF), zinc nitrate hexahydrate ($Zn(NO_3)_2 \cdot 6H_2O$), polyethylene glycol (PEG-20000), triethanolamine ($C_6H_{15}NO_{3,}$ TEA), sodium hydroxide (NaOH), sodium bicarbonate ($NaHCO_3$), potassium peroxydisulfate ($K_2S_2O_8$) and potassium chloride (KCl) were supplied by Sinopharm chemical reagent Co., Ltd. (Shanghai, China). Titanium isopropoxide ($C_{12}H_{28}O_4Ti$, TIP), hexadecylamine ($C_{16}H_{35}N$, HDA) and styrene (C_8H_8) were obtained from Sigma-Aldrich. Benzotrifluride ($C_7H_5F_3$, BTF), was purchased from Alfa Aesar Co., Ltd. (Tianjin, China). All materials were used as received without further treatment. Deionized water was obtained from local sources.

Fabrication of SiO_2 spheres: SiO_2 spheres were prepared *via* the Stöber method.[1] In a typical synthesis procedure, TEOS (2 mL) was injected into a mixture of isopropanol (40 mL), H_2O (1 mL) and $NH_3 \cdot H_2O$ (2 mL) at room temperature under magnetic stirring. After reacting for 4 h, the colloidal spheres were collected by centrifugation and washed with H_2O for several times. Then, the sediment was dried in an electronic oven at 60 °C. The diameter of SiO_2 sphere was tuned by changing the concentration of $NH_3 \cdot H_2O$ with other conditions unchanged.

Fabrication of ZnO spheres: $Zn(NO_3)_2 \cdot 6H_2O$ (0.45 g) was dissolved in H_2O (80 mL), and then PEG-20000 (1 g) and TEA (2 mL) were introduced sequentially with stirring to form a clear solution. The mixed solution was transferred into a Teflon-lined autoclave. Then the autoclave was sealed into a stainless steel tank and maintained at 130 °C for 2 h without shaking or stirring. After the autoclave had been cooled to room temperature naturally, white crystalline products were harvested by centrifugation and thorough washings with DI water several times. Finally, the product was dried at 60 °C in an oven for further preparations.[2, 3]

Fabrication of polystyrene (PS) spheres: Monodisperse polystyrene spheres (PS) were prepared by dispersion polymerization. In brief, all of styrene (7 mL), NaOH (0.048 g), $NaHCO_3$ (0.048g) and H_2O (130 mL) were added into a 250 mL four-necked round-bottom flask equipped with a mechanical stirrer, a thermometer with a temperature controller, a N_2 inlet, a Graham condenser and a heating mantle. The solution was deoxygenated by bubbling N_2 at room temperature for 60 min. Then, the solution was heated to 75 °C and $K_2S_2O_8$ (0.13 g in 5 mL H_2O) was added. The reaction was maintained for 24 h while stirring at 250 rpm. The obtained dispersion was centrifuged, washed with ethanol, and finally, dried in vacuum at room temperature for 24 h.[4-6]

Fabrication of TiO_2 spheres: In a typical synthesis, HDA (1.75 g) was dissolved in 200 mL absolute ethanol, followed by the addition of 0.80 mL KCl solution (0.1M) and 0.51 mL of H_2O. The mixture was stirred at room temperature in a conical flask. While stirring, TIP (4.32 mL) was added in one step. After that, the resulting solution was kept static for 10h. A white substance was formed. The suspension was centrifuged at 3000 rpm for 2 min and washed with ethanol. The cycles of separation and washing were repeated three times. The washed samples were then dried at 60 °C for further use.[7, 8]

Fictionalization of spherical supports: To functionalize the surface of SiO_2 spheres with positive charge, SiO_2 (0.4 g) spheres were first dispersed in 200 mL of ethanol by sonication for 30 min,

followed by addition of 2 mL of APTES. Then, the mixture was kept at 60 °C for 4 h with stirring. Afterwards, APTES-treated SiO_2 spheres were sufficiently rinsed with ethanol to remove the remaining APTES moiety and then dried at 60 °C in an electronic oven for further use.[9, 10] APTES-functionalized ZnO, PS and TiO_2 samples were synthesized through the same procedures as APTES-treated SiO_2 except that the blank SiO_2 spheres were replaced by ZnO, PS and TiO_2, respectively.

Synthesis of citrate-stabilized metal nanoparticles (NPs): Citrate-stabilized metal NPs were synthesized according to the previous reports with some modification.[9, 11-13] Typically, 26 mL of sodium citrate aqueous solution (2.8 mM) was added to 50 mL $HAuCl_4 \cdot 3H_2O$ aqueous solution (0.4 mM) at room temperature. The mixture was stirred to blend well and then 5 mL of $NaBH_4$ aqueous solution (12 mM) was added dropwise with vigorous stirring. After kept stirring for 2 h, the as-obtained metal colloidal nanoparticles should be stored in a refrigerator at 4 °C for further use.[12, 13] Ag and Pt NPs were synthesized through the similar procedures except that $HAuCl_4 \cdot 3H_2O$ was replaced by $AgNO_3$ and $H_2PtCl_6 \cdot 6H_2O$ aqueous solution.[9, 11]

Synthesis of supported metal NPs composites: The supported metal NPs composites were prepared by electrostatic self-assembly method.[9, 10, 14] In a typical preparation procedure, the calculated amount of the Au colloids was added to APTES-modified SiO_2 aqueous suspension (0.5 mg/mL) to prepare Au-SiO_2 with x% weight content of Au NPs named x% Au-SiO_2. The mixing solution was stirred vigorously for 2 h to obtain a suspension. Then, this suspension was washed with H_2O for several times and then fully dried at 60 °C in an oven. The preparation procedures of other supported metal NPs composites were the same as the preparation of Au-SiO_2 composites using the corresponding metal NPs and spherical supports for the self-assembly.

Preparation of the Au-SiO_2@TiO_2 composites: For coating of TiO_2 on Au-SiO_2, 0.04 g of as-prepared x% Au-SiO_2 composites was homogeneously dispersed in 9.74 mL of ethanol by ultrasonication, followed by the addition of 0.08 g of HDA and 0.2 mL of ammonia, and stirred at room temperature for 1 min to form uniform dispersion. Then, 1 mL of TIP ethanol solution (0.05 mL TIP disperse in 2 mL ethanol) was added to the dispersion under stirring. After reaction for 10 min, the product Au-SiO_2@TiO_2 nanostructures were collected by centrifugation and then washed several times with H_2O and ethanol. In this case, the TiO_2 shell thickness of the sample is about 11 nm named x% Au-SiO_2@TiO_2 with medium-thickness shell. The TiO_2 shell thickness could be varied by changing the volume of additional TIP ethanol solution.[15] When the amounts of TIP ethanol solution is increased to 2 mL, the TiO_2 shell thickness is changed to 23 nm. Further decrease of the added amount of TIP ethanol solution to 0.5 mL results in product with TiO_2 shell around 6 nm. The as-prepared catalysts were further treated with 60% $HClO_4$ under ultrasonic condition for 10 min, then thoroughly washed with H_2O and dried at 60 °C for further photoactivity test.[16, 17]

Preparation of the Au@TiO_2 composites: 50 mg Au-SiO_2@TiO_2 was dispersed in 25 mL H_2O, and then 1 mL NaOH (2.5 M) was dropped into the suspension under sonication for 30 min. After etching for 24 h, the Au@TiO_2 composites were formed. The resulting sample was collected by centrifugation, washed with ethanol, and dried at 60 °C for further use.[18]

Photocatalytic oxidation of benzylic alcohols: A mixture of alcohol (0.1 mmol) and photocatalyst (20 mg Au-SiO_2 or 25 mg core-shell Au-SiO_2@TiO_2) was dispersed in oxygen-saturated benzotrifluoride (BTF; 1.5 ml). The mixture was transferred into a 10 ml Pyrex glass bottle filled with molecular oxygen at a pressure of 0.1 MPa and stirred for 1 h to blend the catalyst evenly into the solution. The suspensions were irradiated with visible-near infrared (vis-NIR, 410 nm $< \lambda <$ 1100 nm)

light. After reaction, the product and remaining substrate were analyzed with an Agilent Gas Chromatograph (GC-7820). Conversion (%) of the benzylic alcohols during the photocatalytic reaction is reported as $(C_0\text{-}C)/C_0 \times 100\%$, where C_0 is the initial concentration of reactant after the establishment of adsorption desorption equilibrium, and C is the concentration of the reactant after the photocatalytic reaction.

Photocatalytic reduction of CO_2: The photocatalytic reduction of CO_2 was performed in a gas-solid heterogeneous reaction mode in a quartz reactor (volume, ~25ml). The loading mass of the photocatalyst sample was 20 mg Au-SiO_2 (or 25 mg core-shell Au-SiO_2@TiO_2). This system was evacuated by a mechanical pump and filled with pure CO_2 gas. The evacuation-filling operation was repeated five times. 0.5ml of evacuated water was introduced finally with a syringe via the septum into the sample cell (5 ml) hanging in the quartz reactor. The reactor was kept in dark for 1 h to establish the adsorption-desorption equilibrium and then irradiated with vis-NIR (410 nm < λ < 1100 nm) light. The temperature of the reactor was kept at 25 °C with an electronic fan. At certain irradiation time interval during the experiment, 1ml of reactive gas was taken from the reactor with a syringe for analysis. The amounts of the products were analyzed by gas chromatography (GC 2014C) fitted with a high-sensitivity thermal conductivity detector (TCD) detector, a flame ionization detector (FID) detector and a methanation reactor. After the effluents were separated by the PN pre-column and 5A molecular sieve column, H_2 and O_2 were analyzed by the TCD detector. CH_4 was analyzed by the FID detector. CO was further converted to CH_4 by a methanation reactor and then analyzed by the FID detector. Argon was used as the carrier gas.

***Photocatalytic water splitting**:* Photocatalytic water splitting to H_2 was carried out in a Pyrex top-irradiation reaction vessel connected to a glass closed gas circulation system. H_2 evolution analysis was performed by dispersing 20 mg Au-SiO_2 (or 25 mg core-shell Au-SiO_2@TiO_2) catalyst powder in an aqueous solution (72 ml) containing lactic acid (8 ml) as the sacrificial electron donor. The reactant solution was evacuated several times to remove air completely before the photocatalytic reaction, and then irradiated with visible-near infrared (vis-NIR, 410 nm < λ < 1100 nm) light. The temperature of the reactant solution was maintained at 5 °C by a flow of cooling water during the reaction. The evolved gases were analyzed by an online gas chromatograph (Shimadzu GC-2014C, MS-5 A column, Ar carrier) equipped with a TCD.

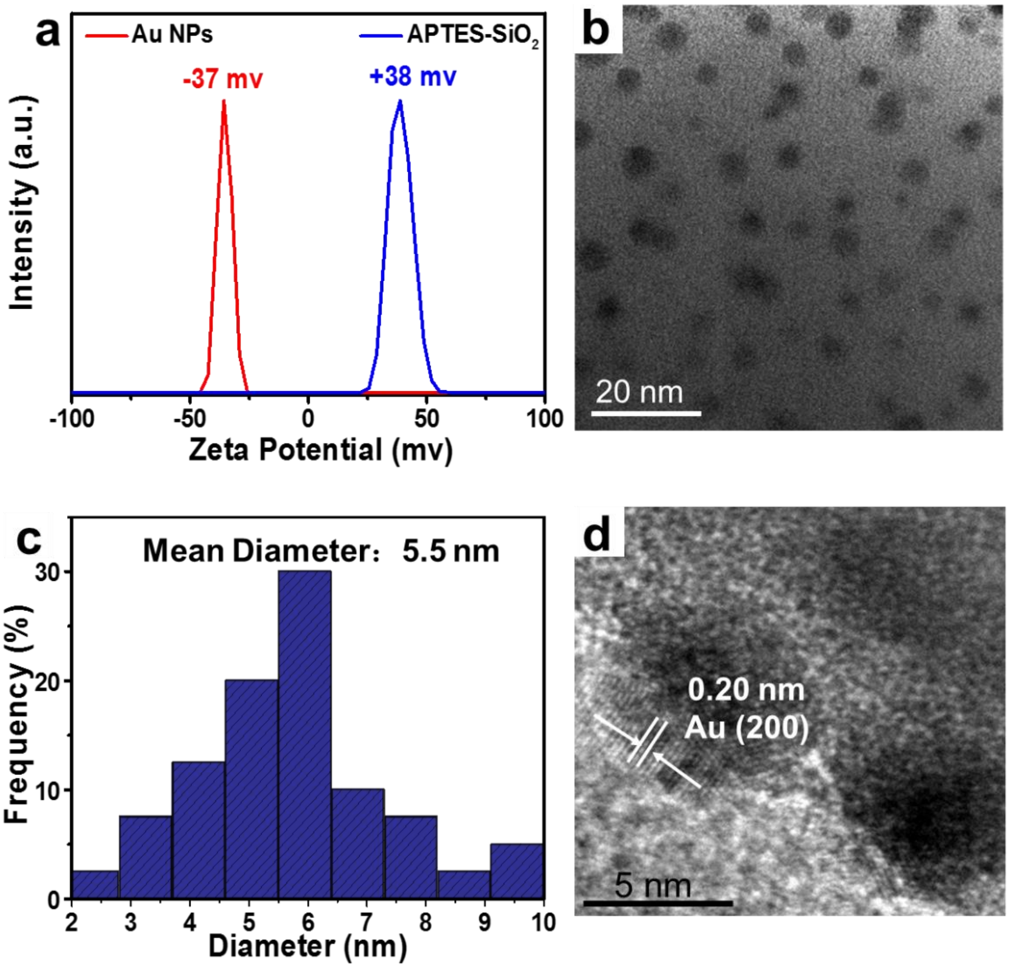

Fig. S1 (a) Zeta potential of the Au nanoparticles (NPs) and 3-aminopropyltriethoxysilane-modified SiO_2 spheres (APTES-SiO_2). (b) Transmission electron microscopy (TEM) image of 5% Au-SiO_2. (c) Size distribution histogram of Au NPs corresponding to (b). (d) High-resolution TEM image of the 5% Au-SiO_2 composite.

Note: As shown in **Fig. S1a**, after functionalization with 3-aminopropyltriethoxysilane (APTES), the Zeta potential of SiO_2 is positively charged. Whereas, the Zeta potential analysis on Au NPs dispersion reveals a negatively charged surface. Thus, it is reasonable that the positively charged APTES-functionalized SiO_2 and negatively charged Au NPs colloid can spontaneously establish a solid basis of electrostatic attraction for the self-assembly construction of Au-SiO_2 composite.

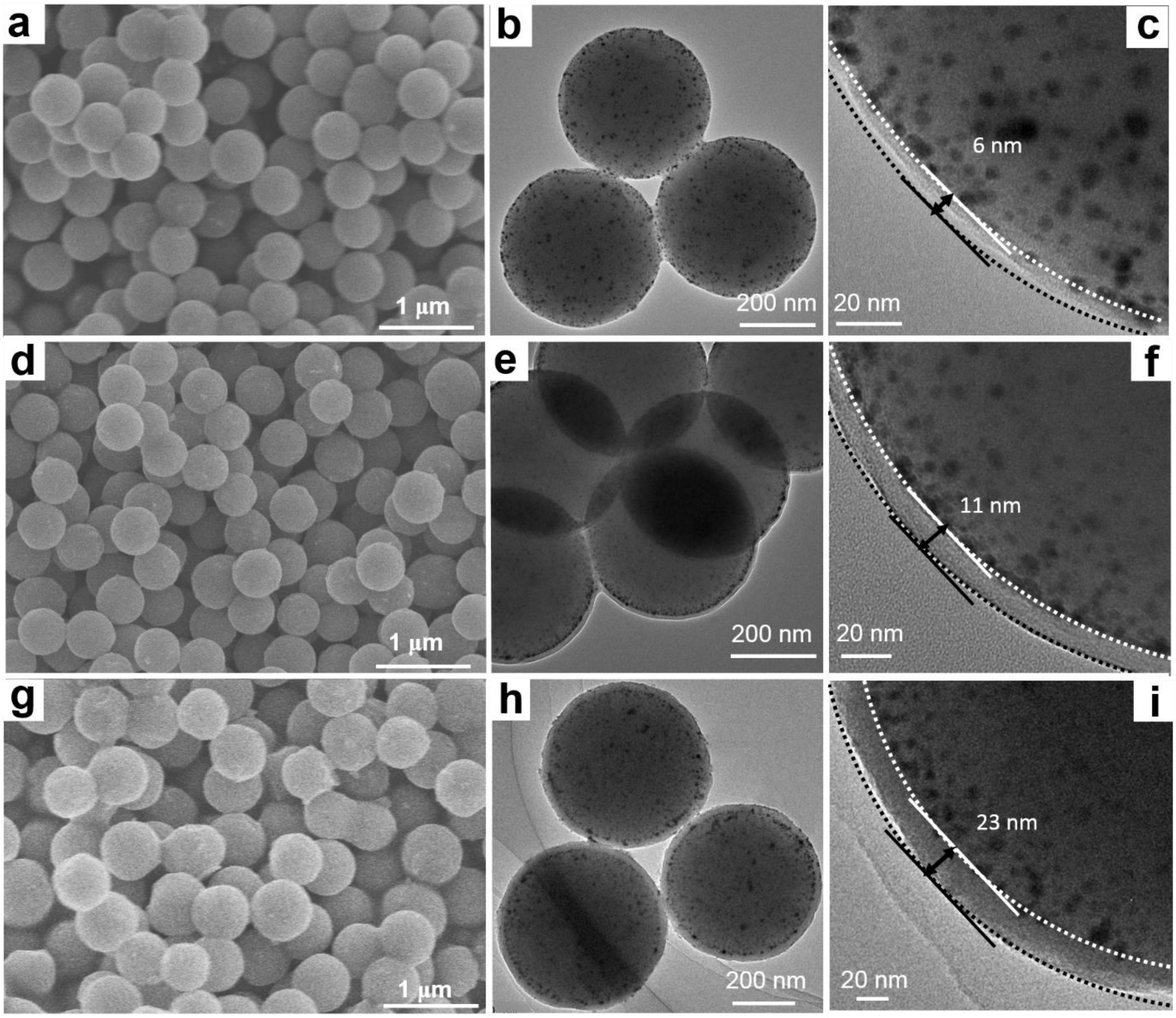

Fig. S2 (a) Scanning electron microscopy (SEM) image and (b and c) transmission electron microscopy (TEM) images of 5% Au-SiO_2@TiO_2 with TiO_2 shell thickness of 6 nm. (d) SEM image and (e and f) TEM images of 5% Au-SiO_2@TiO_2 with TiO_2 shell thickness of 11 nm. (g) SEM image and (h and i) TEM images of 5% Au-SiO_2@TiO_2 with TiO_2 shell thickness of 23 nm.

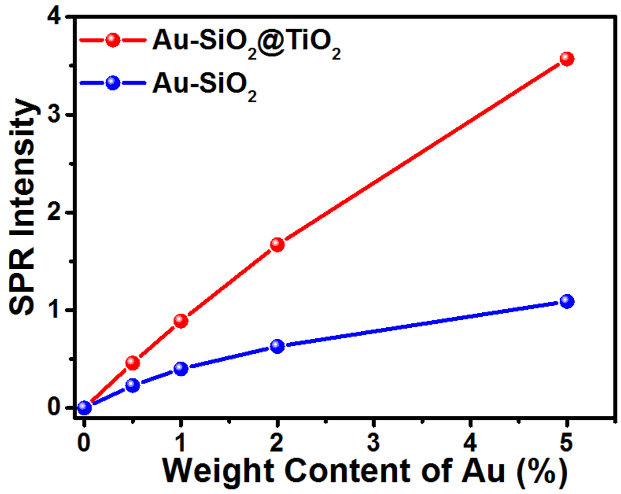

Fig. S3 Plot of surface plasmon resonance (SPR) absorption intensity of Au-SiO_2 and 5% Au-SiO_2@TiO_2 with TiO_2 shell thickness of 11 nm versus weight loading of Au NPs.

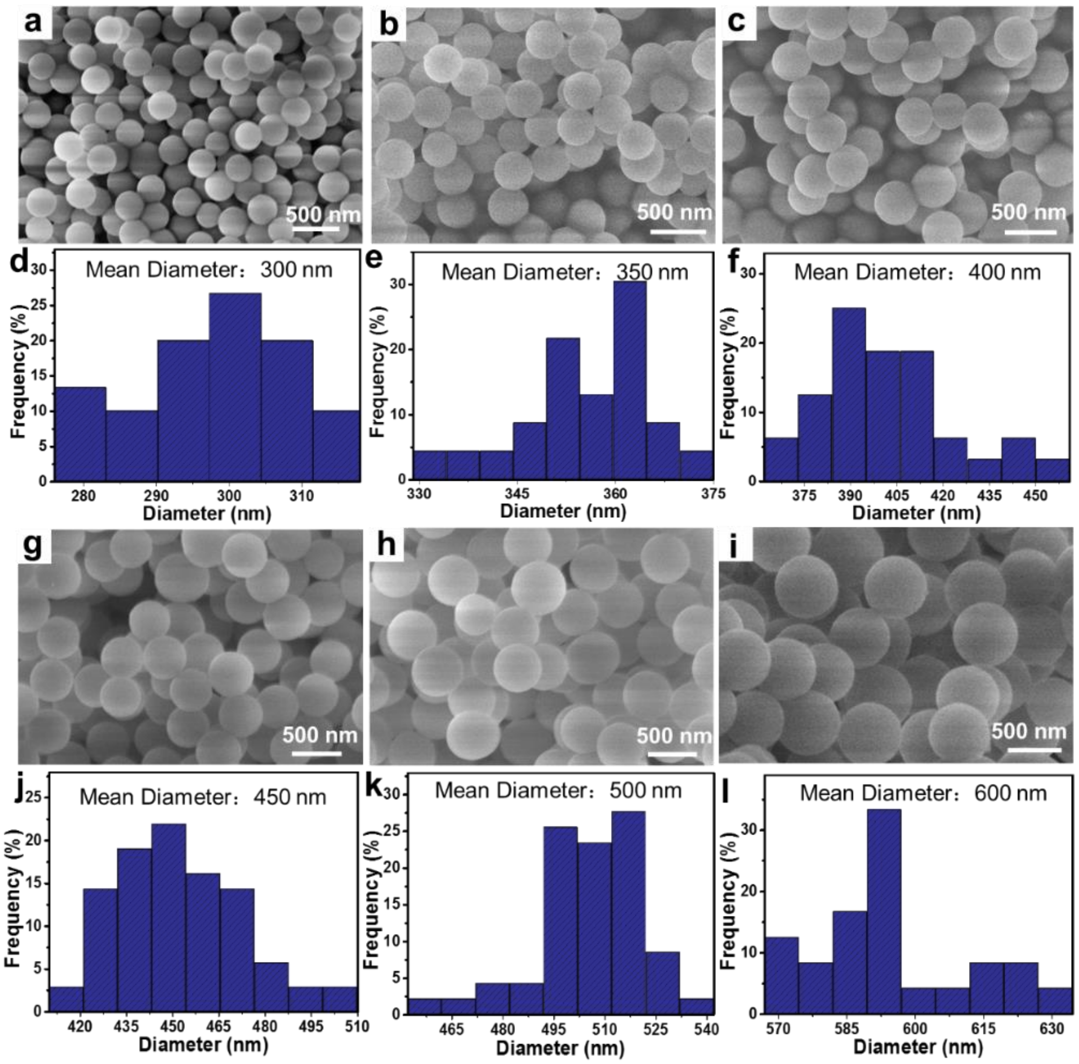

Fig. S4 Scanning electron microscopy (SEM) images of SiO_2 spheres with different average diameters of (a) 300 nm, (b) 350 nm, (c) 400 nm, (g) 450 nm, (h) 500 nm, (i) 600 nm and (d-f and j-l) the corresponding diameter distribution histograms.

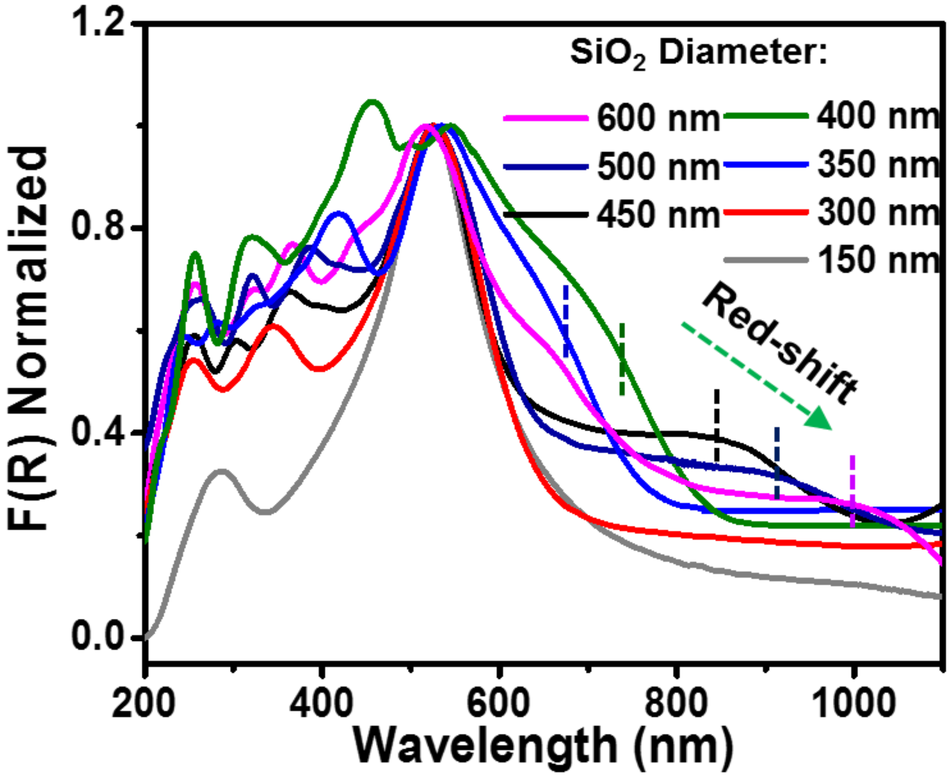

Fig. S5 Ultraviolet-visible-near infrared (UV-vis-NIR) diffuse reflectance spectra (DRS) of 1% Au-SiO_2 with different sizes of SiO_2 spheres.

Note: In order to investigate the possible effect of SiO_2 size on the optical property of the supported Au NPs, we also prepared other five different sizes of SiO_2 supports (see **Fig. S4**) with the average diameter range from 300 to 600 nm, to synthesize Au-SiO_2 composites. It can be seen from the DRS results in **Fig. S5** that small-sized SiO_2 spheres (with the diameter < 350 nm) have only affected the photoabsorption of Au NPs in the ultraviolet (UV) region. As the size of SiO_2 sphere becomes larger, more absorption modes are observed within the UV-vis-NIR region. When the diameter of the SiO_2 is larger than 400 nm, the Au-SiO_2 sample exhibits a new absorption band in the near infrared (NIR) region. In addition, the absorption maxima (marked with dashed line) of Au-SiO_2 show an obvious red-shift along with the increment of SiO_2 diameter.

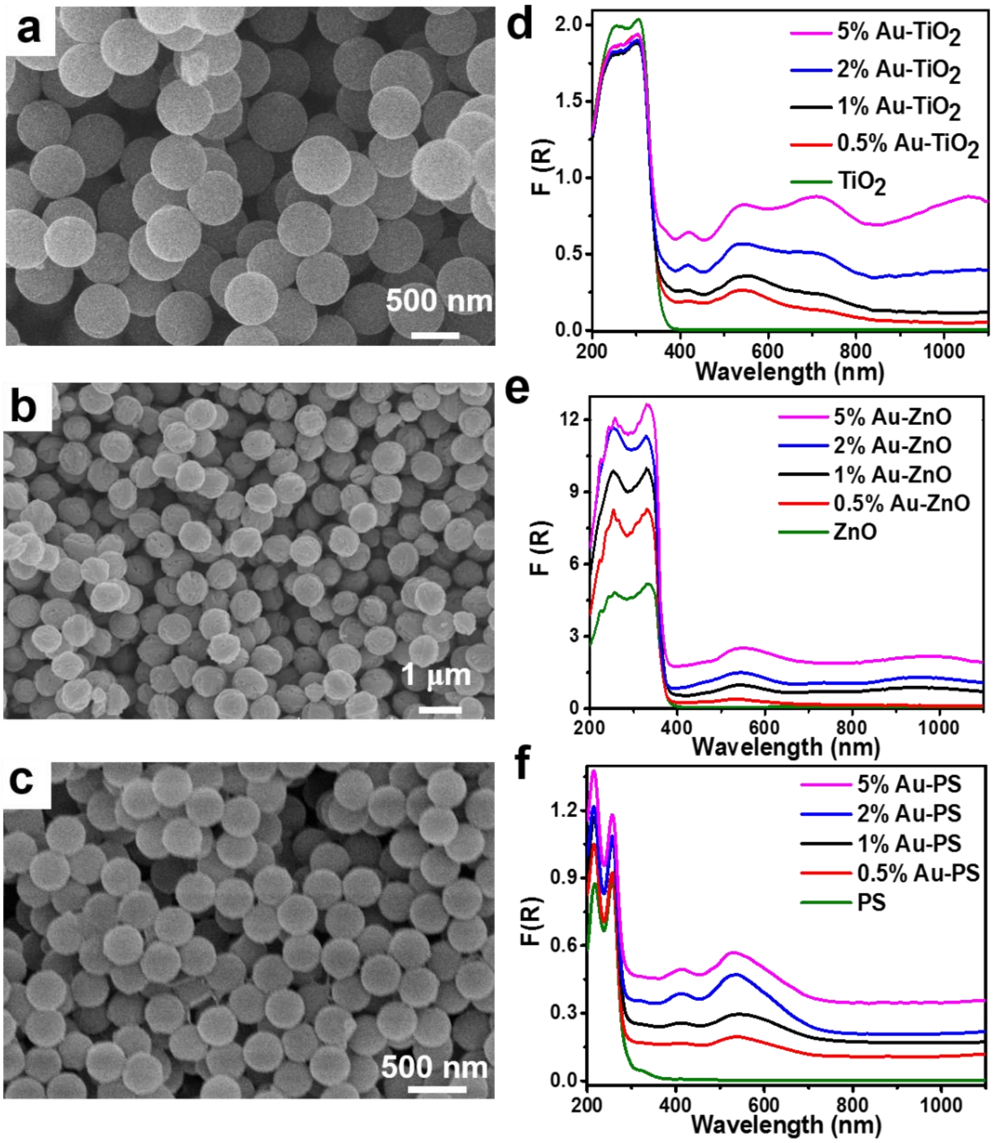

Fig. S6 Scanning electron microscopy (SEM) images of (a) TiO_2 spheres, (b) ZnO spheres and (c) polystyrene (PS) spheres. Ultraviolet-visible-near infrared (UV-vis-NIR) diffuse reflectance spectra (DRS) of (d) Au-TiO_2 samples, (e) Au-ZnO samples and (f) Au-PS samples with different weight content of Au.

Note: **Fig. S6** shows the SEM images of TiO_2, ZnO and PS spheres and the UV-vis-NIR DRS spectra of these sphere supported Au NPs composites. The absorption intensity gradually enhance with the increase of Au NPs loading in these supported Au NPs composites. These DRS results reveal that the constructing of dielectric spheres supported Au NPs composite by self-assembly approach is a facile and universal method to enhance the resonance absorption and extend absorption band of Au NPs.

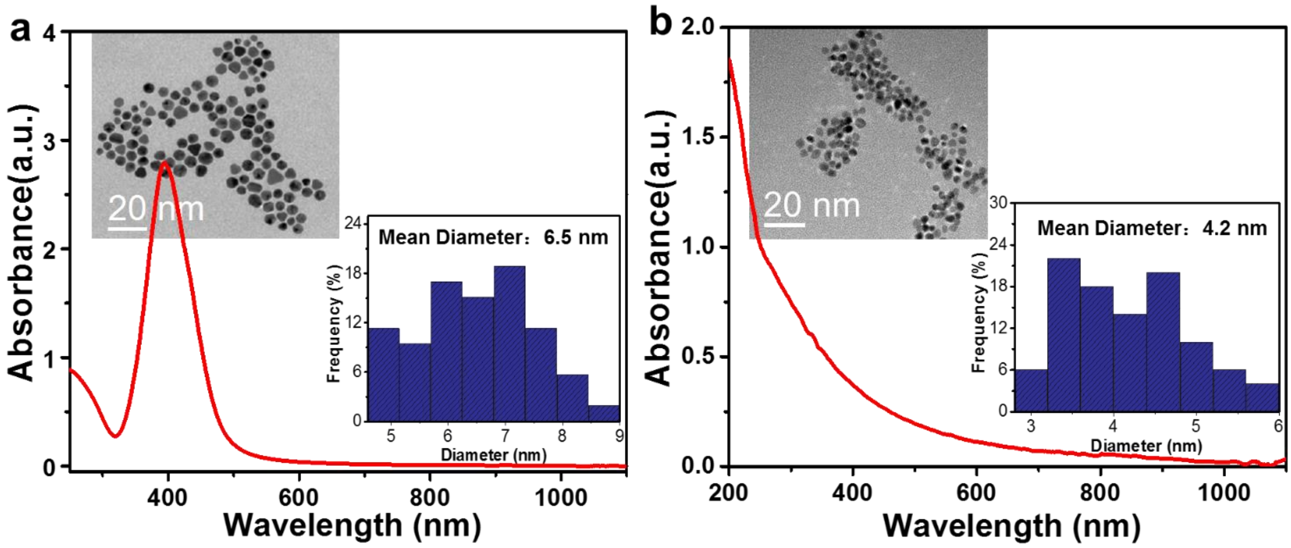

Fig. S7 Ultraviolet-visible-near infrared (UV-vis-NIR) absorption spectrum of (a) Ag nanoparticles (NPs) and (b) Pt NPs. Insets show transmission electron microscopy (TEM) images and size distribution histograms of corresponding metal NPs.

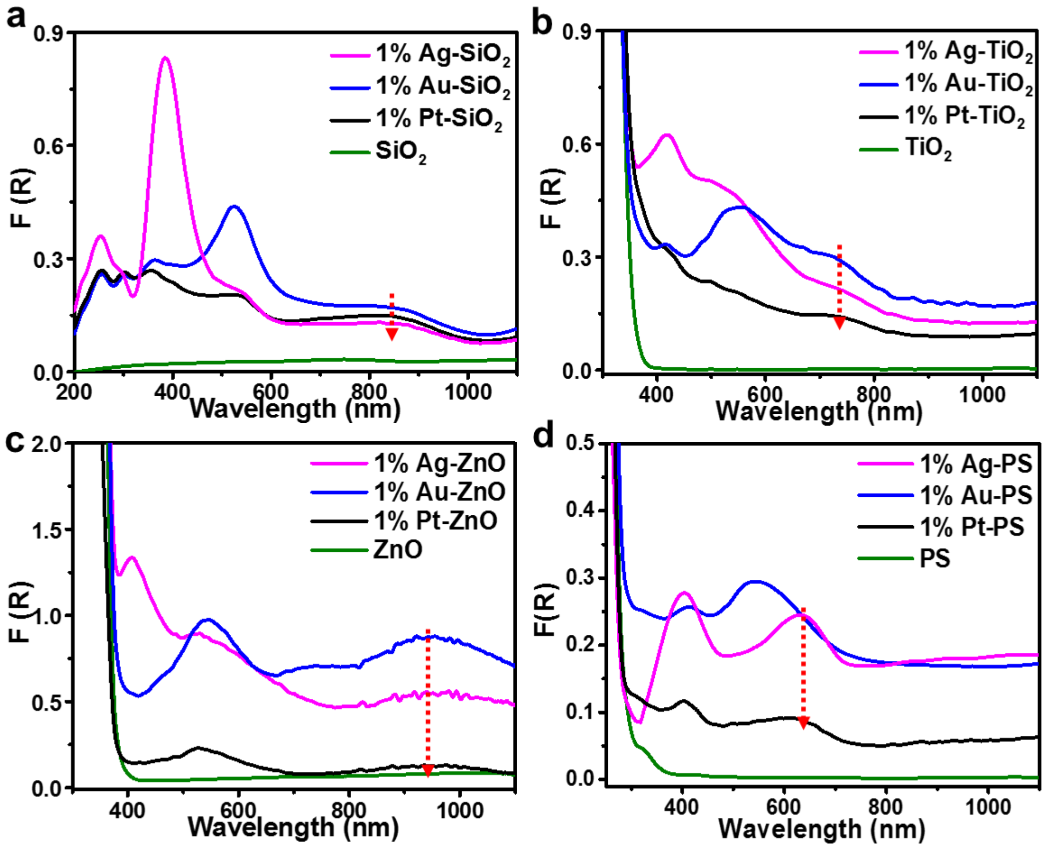

Fig. S8 Ultraviolet-visible-near infrared (UV-vis-NIR) diffuse reflectance spectra (DRS) of (a) SiO_2 and 1% M (M=Au, Ag, Pt)-SiO_2, (b) TiO_2 and 1% M-TiO_2, (c) ZnO and 1% M-ZnO, (d) polystyrene (PS) and 1% M-PS.

Note: **Fig. S8** shows the UV-vis-NIR DRS spectra of dielectric spheres supported metal nanoparticles (NPs) composites. It can be seen that similar to the supported Au NPs, loading Ag and Pt NPs onto different spherical supports (SiO_2, TiO_2, ZnO and PS) can also lead to the observation of distinct absorption peaks in ultraviolet-visible (UV-vis) or near infrared (NIR) region. In addition, no matter what kind of the supported metal NPs is, the peak position of absorption maxima (marked with arrows) almost keeps unchanged for a certain dielectric sphere core, indicating that the near-field scattering-mediated absorption enhancement closely depends on the property of the dielectric sphere.

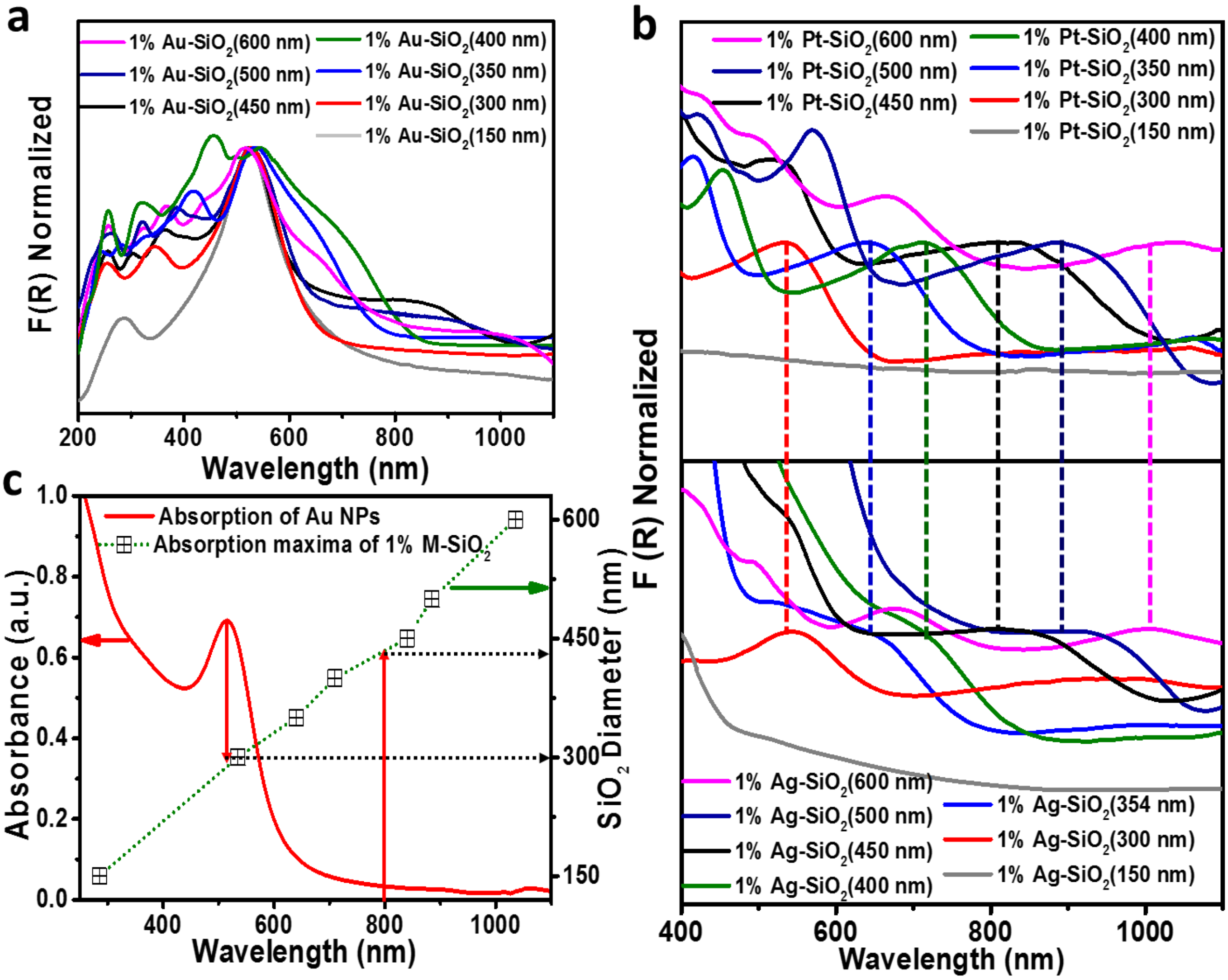

Fig. S9 Ultraviolet-visible-near infrared (UV-vis-NIR) diffuse reflectance spectra (DRS) of (a) 1% Au-SiO_2, (top of b) 1% Pt-SiO_2 and (bottom of b) 1% Ag-SiO_2. (c) Absorption spectra (red curve) of Au nanoparticles (NPs) and absorption maxima of 1% M (M=Au, Ag, Pt)-SiO_2 with different diameter of SiO_2 spheres. The numbers in brackets of (a and b) represent the diameters of different SiO_2 cores.

Note: In the absorption spectrum of Au-SiO_2 (**Fig. S9a**), each fine absorption mode corresponds to a specific photon resonance, but some resonance modes are superimposed with the intrinsic surface plasmon resonance (SPR) absorption of Au NPs, thereby making these modes more complicated and not clearly identified, especially in visible light region. For example, in **Fig. S8a**, the near-field scattering-mediated absorption mode in 535 nm is overlapped with the SPR absorption of Au NPs resulting in an averaging sharp resonance mode in 525 nm of 1% Au-SiO_2. However, the peak position of specific absorption enhancement of supported metal NPs is independent of the nature of metal NPs (**Fig. S8**), suggesting that the Ag and Pt NPs as antennas may provide more detailed information for studying the absorption modes of supported metal NPs, as they will reduce the interference from background light absorption of metal NPs in visible light region and enable the discrimination of the near-field scattering-mediated modes. As a proof of concept, we prepared Pt-SiO_2 and Ag-SiO_2 composites with the same diameter of SiO_2 spheres as that in Au-SiO_2. As

expected, it is easier to distinguish near-field scattering-mediated absorption modes from Pt-SiO_2 and Ag-SiO_2 within visible-near infrared (vis-NIR) region (**Fig. S9b**). The variation trends of the absorption maxima with increasing the diameter of SiO_2 are summarized in **Fig. S9c**. As is clearly seen, in order to generate efficient cooperative effect between near-field scattering and SPR absorption of Au NPs, the diameter of SiO_2 spheres should be selected at ~300 nm. With regard to producing distinct near infrared (NIR) light (λ > 800 nm) absorption peaks, the diameter of SiO_2 spheres should be larger than 420 nm.

Table S1 Photocatalytic reduction of a serious of nitroaromatics over the 5% Au-SiO_2 and 5% Au-SiO_2@TiO_2 in the TiO_2 average shell thickness of 11 nm under visible-near infrared (vis-NIR) (410 nm $< \lambda <$ 1100 nm) irradiation.

Entry	Substrate	Product	Time (min)	Conversion (%)
1	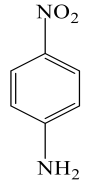	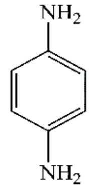	120	92(29)
2	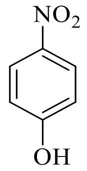	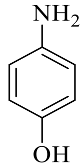	120	99(67)
3	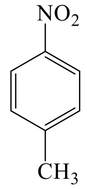	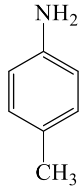	90	84(63)
4	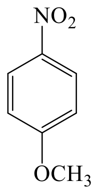	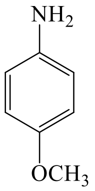	90	90(67)
5	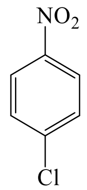	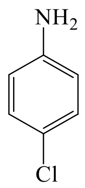	60	91(69)
6	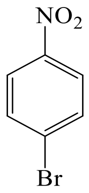	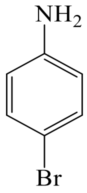	60	90(67)

Note: Data in parentheses are the conversion over 5% Au-SiO_2 sample.

Table S2 Photocatalytic oxidation of a range of benzylic alcohols over the 5% Au-SiO_2 and 5% Au-SiO_2@TiO_2 in the TiO_2 average shell thickness of 11 nm under visible-near infrared (vis-NIR) light (410 nm < λ < 1100 nm) irradiation.

Entry	Substrate	Product	Time (min)	Conversion (%)
1	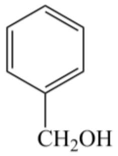	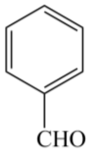	240	25(18)
2	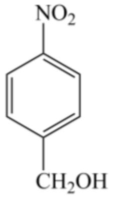	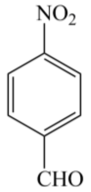	240	44(32)
3	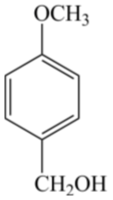	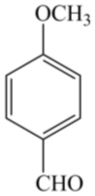	240	42(34)
4	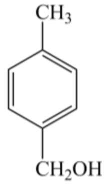	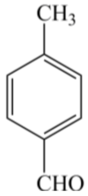	240	28(20)
5	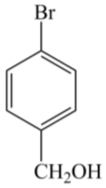	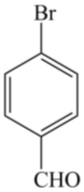	240	39(28)
6	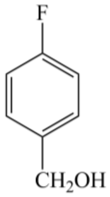	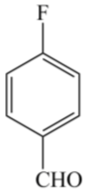	240	31(22)

Note: Data in parentheses are the conversion over 5% Au-SiO_2 sample.

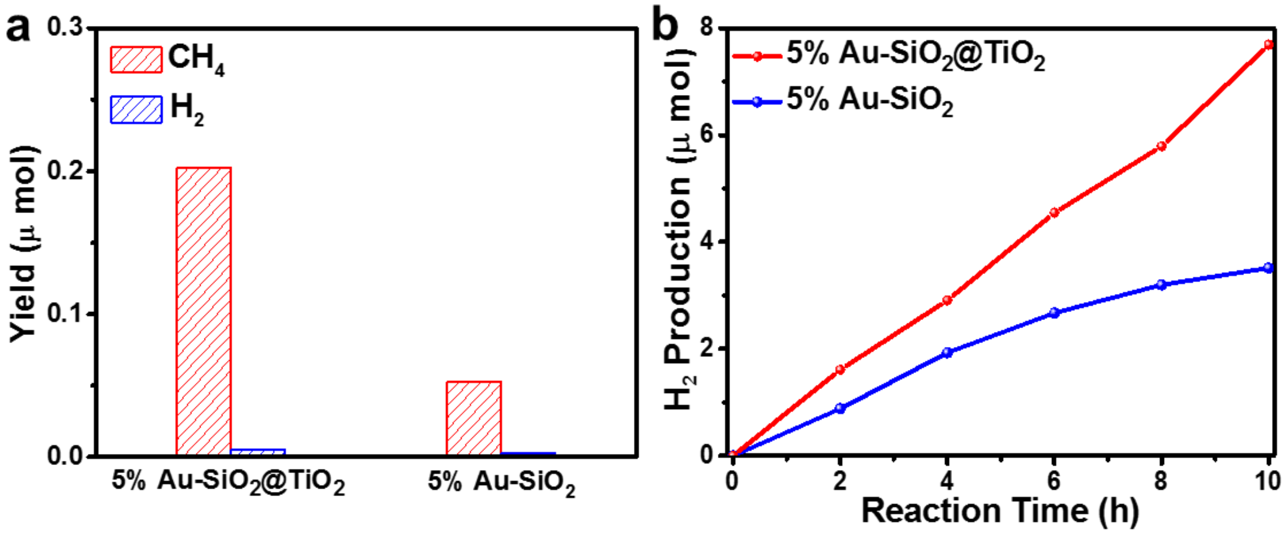

Fig. S10 Photocatalytic (a) reduction of CO_2 and (b) water splitting over the 5% Au-SiO_2 and 5% Au-SiO_2@TiO_2 under visible-near infrared (vis-NIR) light (410 nm < λ < 1100 nm) irradiation. The irradiation time in (a) is 10 h

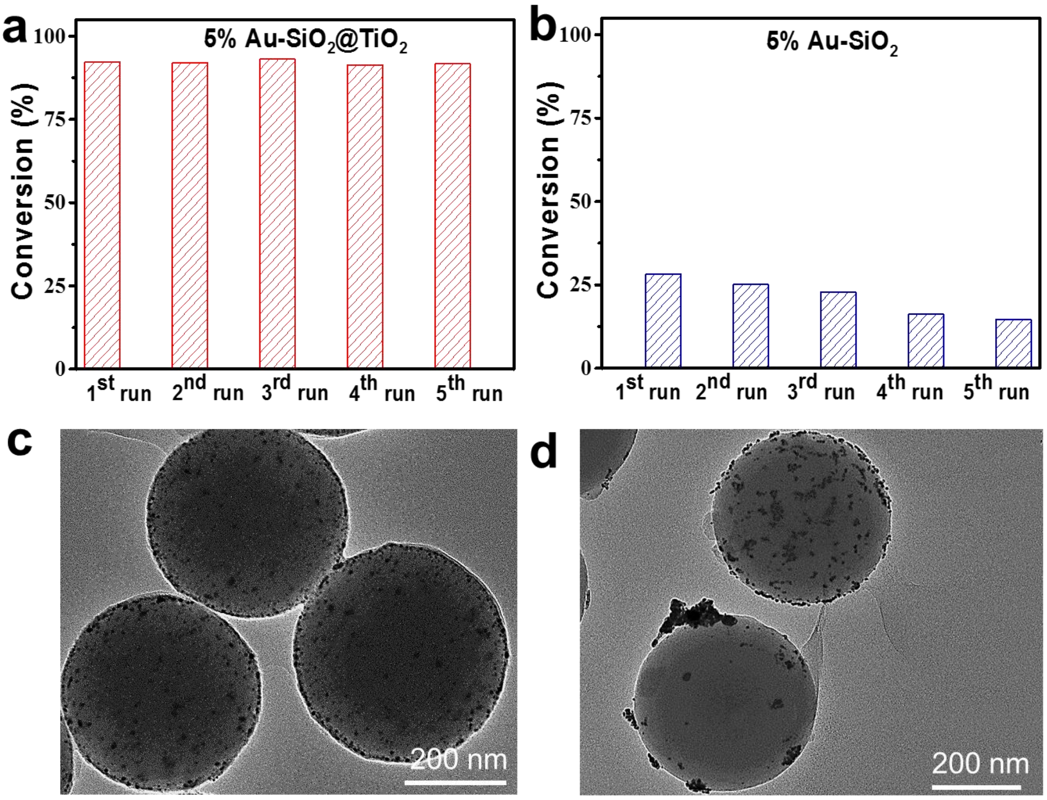

Fig. S11 Recycling tests for photocatalytic reduction of 4-nitroaniline (4-NA) over (a) 5% Au-SiO_2@TiO_2 with TiO_2 shell thickness of 11 nm and (b) 5% Au-SiO_2 under visible-near infrared (vis-NIR) light (410 nm < λ < 1100 nm) irradiation. Transmission electron microscopy (TEM) images of (c) 5% Au-SiO_2@TiO_2 with TiO_2 shell thickness of 11 nm and (d) 5% Au-SiO_2 after recycling photocatalytic reactions.

Note: To evaluate the photostability and reusability of as-prepared samples, recycling tests for

photoreduction of 4-NA were performed. As displayed in **Fig. S11a** and **11b**, compared with 5% Au-SiO_2 photocatalyst, the 5% Au-SiO_2@TiO_2 shows no apparent activity loss over five successive recycles, suggesting that the composites are able to serve as stable photocatalysts under the present reaction conditions. TEM images show that the core-shell structure of 5% Au-SiO_2@TiO_2 is very well reserved after the photocatalytic reaction (**Fig. S11c**). However, severe aggregation and leaching of the Au NPs occurred in used 5% Au-SiO_2 sample (**Fig. S11d**) leading to their obvious deactivation during the recycling reaction.

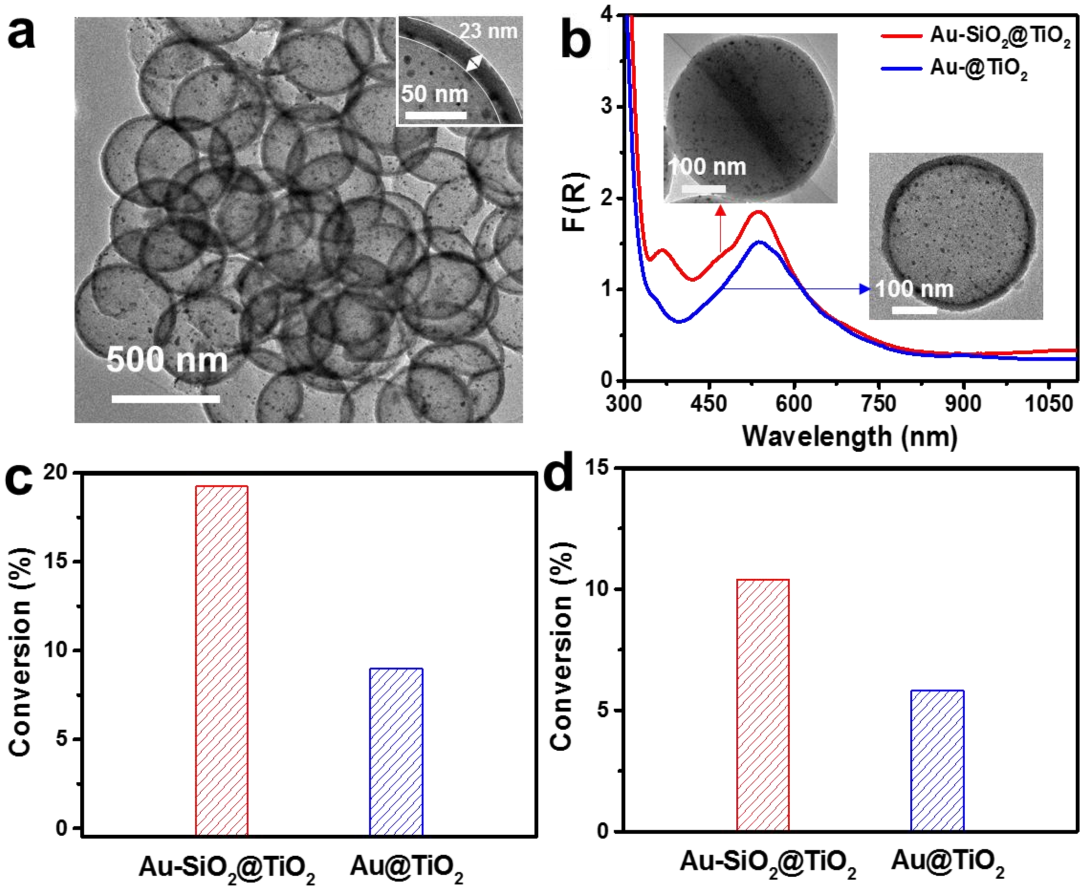

Fig. S12 (a) Transmission electron microscopy (TEM) image of Au@TiO_2 with TiO_2 shell thickness of 23 nm. Inset shows the high magnification TEM image. (b) Ultraviolet-visible-near infrared (UV-vis-NIR) diffuse reflectance spectra (DRS) of Au-SiO_2@TiO_2 and Au@TiO_2 with TiO_2 shell thickness of 23 nm. Insets show the TEM images of corresponding samples. Photocatalytic performance of Au-SiO_2@TiO_2 and Au@TiO_2 with TiO_2 shell thickness of 23 nm for (c) reduction of 4-nitroaniline (4-NA) and (d) oxidation of benzyl alcohol (BA) under vis-NIR light (410 nm $< \lambda <$ 1100 nm) irradiation. The irradiation times in (c and d) are 2 h and 4 h, respectively.

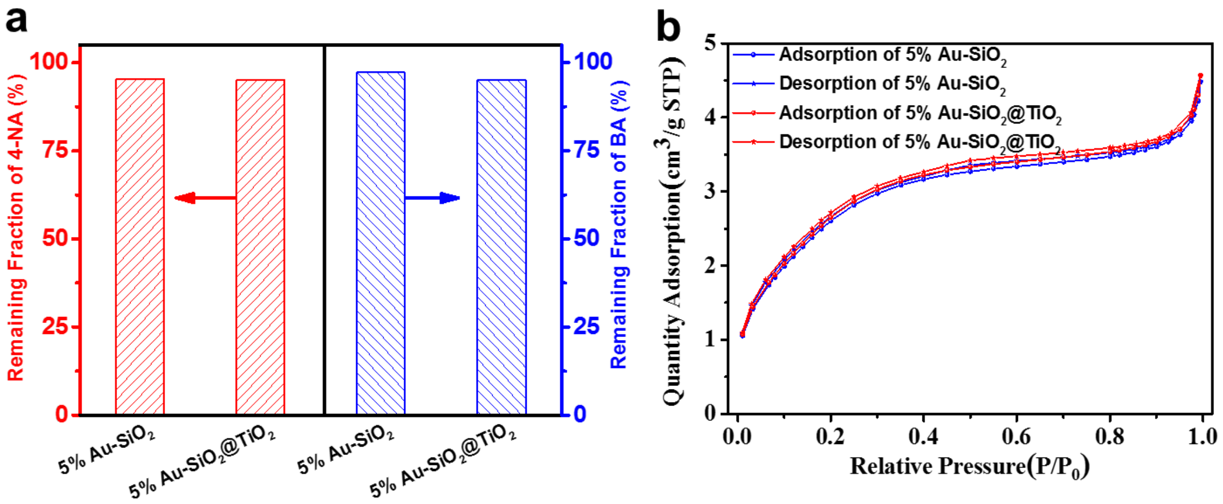

Fig. S13 (a) Column plots showing the remaining 4-nitroaniline (4-NA, left) and benzyl alcohol (BA, right) in solution after being kept in the dark for 1 h until adsorption equilibrium over the 5% Au-SiO_2 and 5% Au-SiO_2@TiO_2 in the TiO_2 average shell thickness of 11 nm. (b) Nitrogen adsorption-desorption isotherms of the 5% Au-SiO_2 and 5% Au-SiO_2@TiO_2 in the TiO_2 average shell thickness of 11 nm.

Table S3 Summary of surface area and pore volume of the 5% Au-SiO_2 and 5% Au-SiO_2@TiO_2 in the TiO_2 shell thickness of 11 nm.

Samples	S_{BET} (m^2/g)	Total pore volume (cm^3/g)
5% Au-SiO_2@TiO_2	10.4	0.006
5% Au-SiO_2	10.2	0.006

Note: The adsorption experiments, surface area and porosity analysis of the samples were performed. The adsorption experiments performed in the dark for 4-NA and BA (**Fig. S13a**) reveal that the presence of TiO_2 shell has no significant effect on adsorptivity of 5% Au-SiO_2. Both Au-SiO_2 and Au-SiO_2@TiO_2 samples have low adsorption capacity toward 4-NA and BA. The surface area and porosity analysis results in **Fig. S13b** and **Table S3** also show that the coating of TiO_2 shell on Au-SiO_2 leads to negligible variation of surface area and pore volume between Au-SiO_2@TiO_2 and Au-SiO_2. Therefore, the specific surface area and adsorption capacity of the samples are not the primary factors accounting for the photoactivity enhancement of Au-SiO_2@TiO_2 as compared to Au-SiO_2.

Table S4 Lifetime (τ_i) and amplitudes (A_i) of transient absorption (TA) decays dynamics for different samples.

Samples	A_1 (%)	τ_1 (ps)	A_2 (%)	τ_2 (ps)
5% Au-SiO_2	94.0	2.5	6.0	126.0
5% Au-SiO_2@TiO_2	92.0	3.4	8.0	292.5

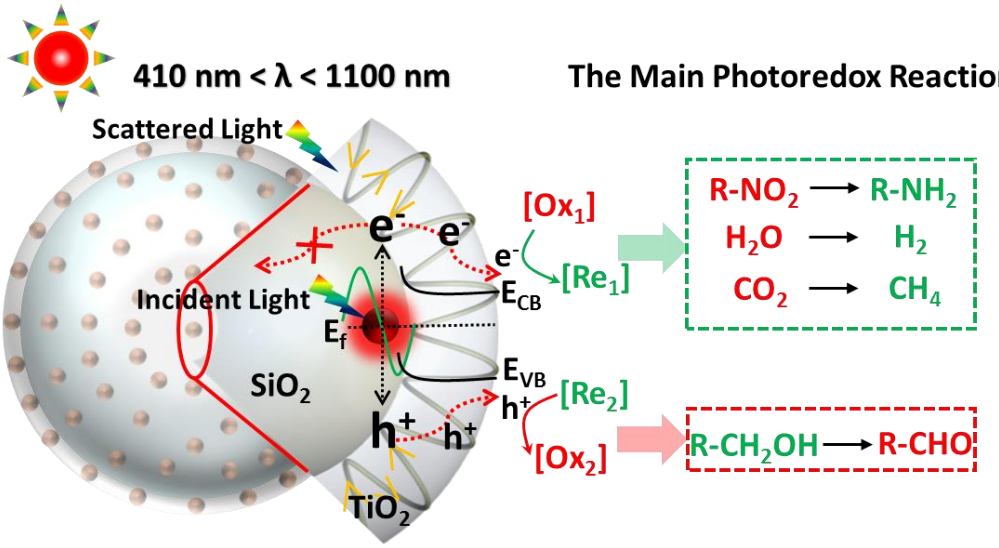

Fig. S14 Schematic illustration for photogenerated charge carriers transfer of Au-SiO_2@TiO_2 under visible-near infrared (vis-NIR) light (410 nm < λ < 1100 nm) irradiation.

Note: Based on the results of controlled experiments, photoelectrochemical (PEC) measurements and transient absorption (TA) spectroscopy, a mechanism for interfacial hot charge carriers generation and transfer involved in photocatalytic redox reactions is proposed, as illustrated in **Fig. S14**. When Au-SiO_2@TiO_2 with core-shell structure is irradiated with vis-NIR light, the Au nanoparticles can simultaneously absorb the incident light and the scattered light at the near-field of the SiO_2 dielectric surface to generate charge carriers. The energetic hot electrons (e^-) are transferred to the conduction band (E_{CB}) of the TiO_2 shell for driving the surface reduction reactions (e.g., anaerobic reduction of aromatic nitro compound, H_2 evolution from water splitting and reduction of CO_2), while the holes (h^+) located below Fermi level (E_f) of Au NPs are tunneled to the surface of TiO_2 to react with the electron donors (e.g., benzylic alcohols and hole scavengers).[9, 19-21]

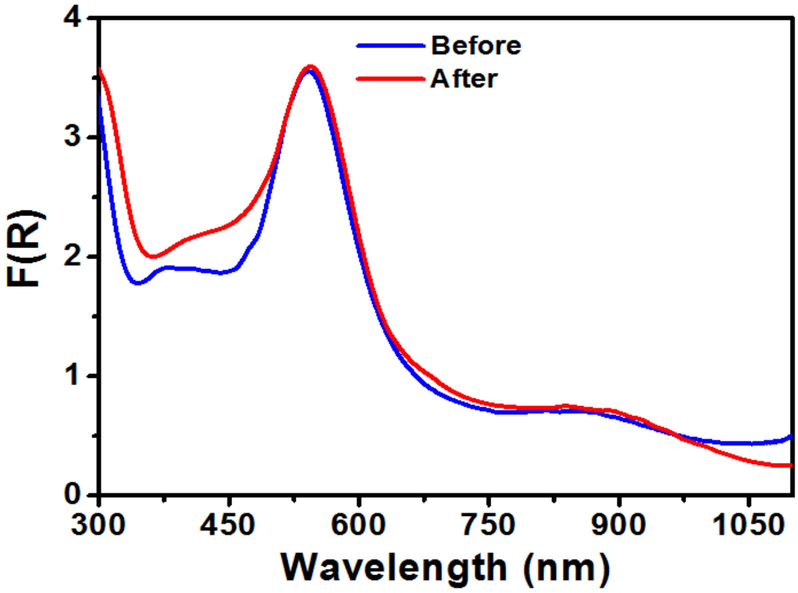

Fig. S15 Ultraviolet-visible-near infrared (UV-vis-NIR) diffuse reflectance spectra (DRS) of 5% Au-SiO_2@TiO_2 with TiO_2 shell thickness of 11 nm before and after photodeposition of Ag.

Note: To further prove the proposed hot electron transfer from photoexcited Au nanoparticles (NPs) to the shell of TiO_2, we performed the control experiment of adding silver nitrate into the aqueous Au-SiO_2@TiO_2 suspension to see whether the photoexcited electrons from Au NPs can reduce silver ions (Ag^+) to metallic Ag particles under visible-near infrared (vis-NIR) light (410 nm < λ < 1100 nm) irradiation.[20, 21] DRS spectrum of 5% Au-SiO_2@TiO_2 after photodeposition of Ag shows clear increased absorption in the range of 300 to 500 nm (**Fig. S15**), which is due to the reduction of Ag^+ to metallic Ag particles on the surface of TiO_2 shell.[20] When Au-SiO_2@TiO_2 is irradiated with vis-NIR light, Au NPs are photoexcited to generate charge carriers. The energetic hot electrons are transferred to the conduction band of TiO_2 shell for Ag^+ reduction, while the holes located below Fermi level of Au NPs can be trapped by the sacrificial agent of ammonium formate.[9, 19-21]

References

1. W. Stöber, A. Fink and E. Bohn, *J. Colloid Interface Sci.*, 1968, **26**, 62-69.
2. S. Xu, Z.-H. Li, Q. Wang, L.-J. Cao, T.-M. He and G.-T. Zou, *J. Alloys Compd.*, 2008, **465**, 56-60.
3. J. Geng, B. Liu, L. Xu, F.-N. Hu and J.-J. Zhu, *Langmuir*, 2007, **23**, 10286-10293.
4. C. E. Reese and S. A. Asher, *J. Colloid Interface Sci.*, 2002, **248**, 41-46.
5. A. Y. Men'shikova, T. G. Evseeva, B. M. Shabsel's, I. V. Balanina, A. K. Sirotkin and S. S. Ivanchev, *Russ. J. Appl. Chem.*, 2005, **78**, 1008-1012.
6. C. E. Reese, C. D. Guerrero, J. M. Weissman, K. Lee and S. A. Asher, *J. Colloid Interface Sci.*, 2000, **232**, 76-80.
7. D. Chen, L. Cao, F. Huang, P. Imperia, Y.-B. Cheng and R. A. Caruso, *J. Am. Chem. Soc.*, 2010, **132**, 4438-4444.
8. C. R. Tubío, F. Guitián, J. R. Salgueiro and A. Gil, *Mater. Lett.*, 2015, **141**, 203-206.
9. N. Zhang, C. Han, Y.-J. Xu, J. J. Foley IV, D. Zhang, J. Codrington, S. K. Gray and Y. Sun, *Nature Photon.*, 2016, **10**, 473-482.
10. Q. Zhang, D. Q. Lima, I. Lee, F. Zaera, M. Chi and Y. Yin, *Angew. Chem. Int. Ed.*, 2011, **50**, 7226-7230.
11. F. Xiao, *J. Phys. Chem. C*, 2012, **116**, 16487-16498.
12. K. R. Brown, D. G. Walter and M. J. Natan, *Chem. Mater.*, 2000, **12**, 306-313.
13. L. Kong, G. Duan, G. Zuo, W. Cai and Z. Cheng, *Mater. Chem. Phys.*, 2010, **123**, 421-426.
14. Y. H. Jang, Y. J. Jang, S. T. Kochuveedu, M. Byun, Z. Lin and D. H. Kim, *Nanoscale*, 2014, **6**, 1823-1832.
15. B. Y. Guan, L. Yu, J. Li and X. W. Lou, *Sci. Adv.*, 2016, **2**, e1501554.
16. Z. Zheng, T. Tachikawa and T. Majima, *J. Am. Chem. Soc.*, 2014, **136**, 6870-6873.
17. L. Liu, S. Ouyang and J. Ye, *Angew. Chem. Int. Ed.*, 2013, **52**, 6689-6693.
18. L. Ilkeun, J. J. Bong, Y. Yadong and Z. Francisco, *Angew. Chem.*, 2011, **123**, 10390-10393.
19. J. Zhang, X. Jin, P. I. Morales-Guzman, X. Yu, H. Liu, H. Zhang, L. Razzari and J. P. Claverie, *ACS Nano*, 2016, **10**, 4496-4503.
20. A. Li, P. Zhang, X. Chang, W. Cai, T. Wang and J. Gong, *Small*, 2015, **11**, 1892-1899.
21. N. Zhou, L. Polavarapu, N. Gao, Y. Pan, P. Yuan, Q. Wang and Q.-H. Xu, *Nanoscale*, 2013, **5**, 4236-4241.
